# Photorelaxation via Water-Mediated Electron Transfer in Fully Solvated Heptazine

**DOI:** 10.1021/acs.jpclett.5c01896

**Published:** 2025-07-31

**Authors:** Laure de Thieulloy, Robson S. Oliboni, Piotr de Silva, Luis G. C. Rego

**Affiliations:** † Department of Energy Conversion and Storage, 5205 Technical University of Denmark, 2800 Kongens Lyngby, Denmark; ‡ Department of Chemistry, 37902 Universidade Federal de Pelotas, Pelotas, RS 96010-900, Brazil; ¶ Department of Physics, Universidade Federal de Santa Catarina, Florianopolis, SC 88040-900, Brazil

## Abstract

Theoretical photochemistry simulations often treat the solvent as static, with only the solute evolving out of equilibrium. However, in polar solvents like water, dynamic solvent effects critically influence photorelaxation. We perform nonadiabatic excited-state molecular dynamics simulations of heptazine (C_6_N_7_H_3_) fully solvated in water and identify a previously unrecognized solvent-mediated photorelaxation pathway. It involves direct hybridization between the 1b_1_ lone pair p-orbitals of water and lone pair orbitals on heptazine nitrogen atoms, bypassing hydrogen bonding. This drives water-to-heptazine electron transfer (ET), forming transient (H_2_O)_*n*_^+^ clusters (2 ≤ *n* ≤ 4) stabilized in hemibonded configurations. The electron–hole pair evolves within a droplet of ∼125 water molecules and recombine on a subpicosecond time scale via back-ET from a pyramidalized aromatic carbon—acting as an intermediate carbanion—to the hemibonded (H_2_O)_2_^+^ cluster. Our findings highlight the critical role of explicit solvent fluctuations and finite-size effects in excited-state dynamics, providing new insights into photorelaxation and radical formation relevant to the photocatalytic cycle of heptazine.

## Graphical abstract

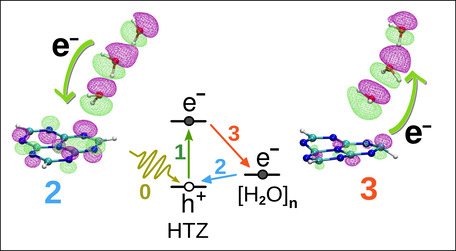

The environment can influence the photorelaxation dynamics of a chromophore either passively or actively.^1^ In the passive case, the environment alters the system’s energy levels without qualitatively modifying the relaxation processes. In this scenario, the medium acts perturbatively, adjusting the energetic conditions without introducing new dynamic pathways. Conversely, an active environment alters chromophore relaxation pathways relative to its isolated behavior. This intrusive role can occur through mechanisms of charge and energy transfer between the chromophore and the medium or by enabling or suppressing specific relaxation pathways.^2^ The Aggregation-Induced Emission (AIE) exemplifies the latter case, wherein aggregation suppresses nonradiative channels and promotes new radiative pathways,^3^ with notable implications for organic optoelectronic devices.^4^ Furthermore, in polar solvents, solvent atomistic dynamics play a crucial role in the chromophore relaxation,^5–7^ impacting fields such as biophysics^8^ and photocatalysis.^9^

Intramolecular relaxation and charge transfer (CT) processes often compete in shaping reaction pathways and determining their efficiencies. For instance, Barbatti^10^ reported that in 7H-adenine within water clusters, solvent-chromophore electron transfer dominates over internal conversion (IC) during S_1_/S_0_ photorelaxation. Similarly, Rego and Bortolini^11^ demonstrated that on semiconductor surfaces functionalized with azobenzene-based chromophores, ultrafast interfacial electron transfer (IET) suppresses the trans → cis photoisomerization of the bound azo-compound. Likewise, Kanai and collaborators^12^ found that ultrafast electron transfer from a molecule to a semiconductor surface inhibits proton transfer within *o*-hydroxybenzaldehyde adsorbed on Si(111) surfaces.

In this work, we investigate the photoinduced excited-state relaxation of the heptazine (HTZ) molecule fully solvated in water using nonadiabatic simulations with quantum-mechanical treatment of large solvation shells. We identify two main relaxation pathways: (i) intramolecular vibrational relaxation, as in the isolated molecule, and (ii) photorelaxation mediated by electron transfer from water to the chromophore.

Heptazine (tri-s-triazine, C_6_N_7_H_3_) is a remarkable aromatic compound that serves as the core unit for various molecular and polymeric materials, self-assembled structures, crystals, and 2D-like organic semiconductors.^13–15^ These compounds, known for their unique electronic structure and stability, have been explored as metal-free photocatalysts for water splitting, hydrogen production, and CO_2_ reduction.^16–19^ Additionally, they have potential applications in photoelectronics, such as light-emitting devices,^13,20^ as heptazine and related compounds exhibit an inversion of the lowest singlet and triplet excited states, violating Hund’s rule.^21–24^

Previous studies on the photorelaxation of solvated heptazine compounds have modeled the solute coupled to only a few water molecules, which does not accurately reproduce the properties of the liquid solvent.^25^ In some cases, these investigations explored the excited-state energies of heptazine–water clusters using static first-principles methods, aiming to elucidate photocatalytic reaction pathways.^7,9,18,26^ These studies predict that upon photoexcitation of HTZ to the first bright π–π* state, an electron is transferred from water to HTZ through a hydrogen-bonded N···H_2_O interaction, followed by hydrogen abstraction from the water molecule, yielding heptazinyl and OH radicals.^9^ However, since these analyses were conducted using a single water molecule or small water clusters, this approach tends to overemphasize the role of hydrogen bonds and neglect the effects of thermal fluctuations present in the liquid phase.

We identify a distinct relaxation mechanism in fully solvated HTZ, involving direct hybridization between the 1b_1_ p-orbitals of water and lone pairs on HTZ nitrogen atoms, without mediation of hydrogen bonds. This enables electron transfer from the valence band maximum of water’s first solvation shell to the HOMO of photoexcited HTZ. In roughly half the trajectories, relaxation proceeds via vibrational modes—primarily ring puckering—with negligible coupling to water. In the rest, full electron transfer occurs, followed by hole delocalization and stabilization through formation of hemibonded (H_2_O)_*n*_^+^ clusters (2 ≤ *n* ≤ 4) with O···O distances below 2.4 Å. The electron–hole pair recombines within a subpicosecond via back-transfer to a pyramidalized carbon, forming an intermediate carbanion. This recombination competes with dissociation of the water cation into H^+^(aq) and OH^•^.

To perform the excited-state quantum dynamics simulations, we implemented an efficient semiempirical version of the Ehrenfest method combined with Coherent Switching with Decay of Mixing (CSDM).^27–31^ A detailed description of this approach, along with its implementation in our semiempirical framework, is provided in the Supporting Information and previous studies.^3,32,33^ CSDM integrates elements of the Ehrenfest method and the Fewest Switches Surface Hopping (FSSH) algorithm into a unified framework. Specifically, it introduces a phenomenological decoherence term into the time-dependent Schrödinger equation, which drives the electronic wave function toward a pointer state associated with a specific potential energy surface (PES), following the concept of pointer states introduced by Zurek.^34,35^ The kinetics of this pointer state is governed by Tully’s fewest switches algorithm.^36^ Unless explicitly stated otherwise, all numerical calculations, including parametrization, geometry optimization, classical molecular dynamics, and excited-state molecular dynamics, were performed using the Dynemol package.^37^

The model system adopted in this study consists of a single heptazine molecule fully solvated by 571 H_2_O molecules within a simulation box under periodic boundary conditions at 300 K and 1 atm. Before combining the solute and solvent in the QM-MM simulations, we independently parametrized the heptazine (C_6_N_7_H_3_) and H_2_O molecules.

To describe the ground-state molecular structure of heptazine, we employed the Amber force field^38^ with GAFF2 parameters (version 2.2.20, March 2021).^39^ Atomic charges were assigned using the AM1-BCC model^40^ within the Antechamber package.^41^ Among the various force field parameters and atomic charge models tested, the combination of GAFF2 parameters with AM1-BCC charges exhibited the best agreement with ground-state geometries obtained from density functional theory (DFT) calculations. To validate the force field, we compared the ground-state optimized geometry of heptazine with DFT results obtained using the long-range corrected hybrid functional wB97xD^42^ and the 6-31g­(d,p) basis set, as implemented in ORCA.^43^ Figures S1 and S2 demonstrate an excellent agreement between the geometries optimized by the classical force field and those obtained from DFT for heptazine; further details are provided in the Supporting Information.

In addition, we parametrized the extended Hückel model Hamiltonian for heptazine (C_6_N_7_H_3_) using Slater-type orbitals (STOs), as detailed in Section 3 of the Supporting Information. For the parametrization, we employed our Adaptive Genetic Algorithm method with Symmetry Descriptors, calibrated to reproduce the charge distribution of the frontier molecular orbitals (MOs) and the excitation energy calculations obtained via algebraic diagrammatic construction to second order, ADC(2), with the cc-pVDZ basis set in TURBOMOLE.^44^ We chose ADC(2) as the reference method for its balance of stability, accuracy, and computational efficiency, as demonstrated by Plasser and collaborators,^45^ who showed that ADC(2) yields consistent results and strong performance in describing the nonradiative decay of the 9H-adenine molecule. ADC(2) is particularly well-suited for modeling dynamical processes within the excited-state manifold. However, caution is warranted in the immediate vicinity of electronic state crossings, where its single-reference character may present limitations. Nonetheless, ADC(2) remains capable of capturing the key puckering deformations responsible for the nonradiative decay pathways of photoexcited 9H-adenine, whose chemical structure closely resembles that of the HTZ molecule. Figures S7 and S8 in the Supporting Information validate the extended Hückel Hamiltonian. Figure S7 demonstrates its accuracy across nuclear distortions, especially near state crossings and puckered geometries. Figure S8 compares excitation energies along Ehrenfest-CSDM trajectories, showing good agreement between the extended Hückel model and ADC(2).

We employed the flexible TIP3P model for water.^46,47^ Given the significance of hydrogen bonding in liquid water, the electronic parametrization for water required the introduction of an extra p orbital to the hydrogen atom of H_2_O to account for polarization effects. The parametrization was conducted using the Adaptive Genetic Algorithm, simultaneously for both the isolated H_2_O molecule (monomer) and a trimer of H_2_O molecules. In this process, we utilized as references the dipole moment and molecular orbital symmetries of the monomer, alongside *ab initio* energies of the trimer obtained using DFT-B3LYP def2-TZVP­(D3BJ). Additionally, we compared the density of states of a simulation box of liquid water with experimental spectra of photoelectron emission to ensure a consistent description of the valence states 1b_2_, 3a_1_, and 1b_1_ of liquid water.^7,48,49^ Figure 1 shows the projected density of states (PDOS) of the HTZ-H_2_O solution averaged over ten uncorrelated system configurations obtained from MM simulations in the ground state after thermalization. The parameters utilized in this study, along with a comparison between the semiempirical methodology and ab initio reference results, are provided as Supporting Information.

**Figure 1:**
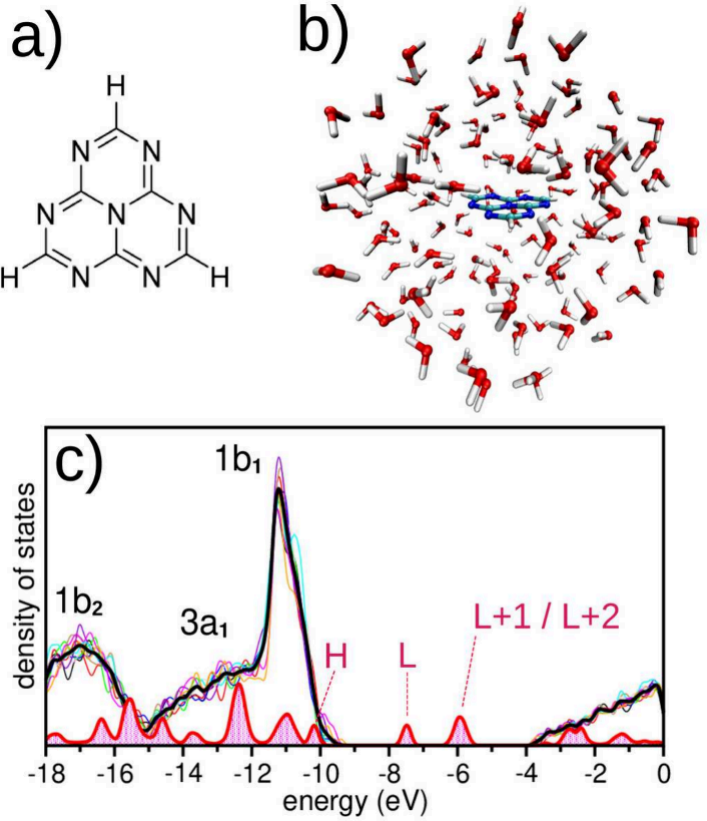
(a) Chemical structure of the heptazine (HTZ) molecule. (b) HTZ solvated in water. The HTZ and water molecules within a 10 Å radius are included in the quantum dynamics simulations. (c) Projected density of states (PDOS) of the HTZ–H_2_O solution, averaged over ten ground-state configurations from thermalized MM simulations. The thick black line shows the averaged PDOS of water; colored lines correspond to individual configurations, with 1b_1_, 3a_1_, and 1b_2_ indicating water valence orbitals. The red curve and background represent the HTZ PDOS, highlighting the HOMO (H), LUMO (L), and the quasi-degenerate L+1 and L+2 orbitals.

During the thermalization and system preparation phases, all molecules were treated as classical objects in the molecular mechanics (MM) simulations. However, for the excited-state nonadiabatic molecular dynamics (NAMD) simulations, the HTZ molecule and water molecules within 10 Å cutoff radius (measured from the central nitrogen of HTZ) are modeled as hybrid quantum-classical objects in the trajectory based framework. In this approach, electrons evolve according to the Ehrenfest-CSDM method, while the nuclei follow molecular mechanics. These definitions are maintained throughout the NAMD simulations. Water molecules initially located outside the cutoff radius are treated as classical objects for the entirety of the simulation. The number of hybrid quantum-classical water molecules in each simulation is approximately 125, depending on the initial system configuration.

After thermalization, a 200 ps ground-state production run in the *NVT* ensemble at 300 K was performed using molecular mechanics (MM). Nuclear positions and velocities were recorded to serve as initial conditions for excited-state nonadiabatic molecular dynamics (NAMD) simulations.

Hereafter, we begin analyze the S_1_ → S_0_ photorelaxation dynamics. Ten trajectories were examined, revealing two distinct relaxation pathways: intramolecular photorelaxation and water-chromophore photorelaxation, both detailed in the following sections. A comprehensive description of all trajectories is provided in the Supporting Information.

The lowest electronic excitation of heptazine has an energy of approximately 2.7 eV in its optimized geometry. Within the one-electron approximation, this excitation can be accurately described as a π → π* transition, involving the HOMO to LUMO electronic states (see Figure S6 for details on electronic transitions). A significant admixture of double excitations, enabling the singlet–triplet inversion in heptazine, is not explicitly accounted for in the model, but the energetic effect is treated empirically through fitting to the ADC(2) energies. Due to the D_3*h*_ symmetry of the molecule, this transition is forbidden under the dipole approximation. However, thermal fluctuations and the presence of the solvent relax this selection rule, allowing photoabsorption to occur, albeit with a weak absorption coefficient.^7^ No direct photoinduced charge transfer between the HTZ and the surrounding water molecules was considered in the model. Thus, in all simulations, the photoabsorption occurs within the HTZ molecule.

We begin by analyzing the photorelaxation of HTZ in trajectories that undergo intramolecular vibrational relaxation, driven by ring puckering distortions with minimal coupling to the solvent. Figure 2 illustrates a representative trajectory, in which the quantum region comprises the heptazine molecule and a droplet of 125 water molecules within a 10 Å cutoff radius, while the remaining water molecules are treated classically. On the left, the figure shows the electronic density of states (DoS) projected onto the quantum H_2_O molecules (gray) and the HTZ molecule (red, scaled by a factor of 4 for clarity). The relevant molecular orbitals (MOs) are labeled, and the vertical green arrow indicates the S_1_ π → π* transition. On the right, the time evolution of MO energies (black lines) is displayed, computed on the fly during the excited-state NAMD simulation. The 1b_1_ band of liquid water forms a mesh of states between −10 and −12 eV, while states at the bottom of the conduction band appear between −2 and −4 eV. The left panel serves as a reference for tracking the MOs of HTZ: the HOMO is located at the edge of the 1b_1_ band, while the LUMO and the LUMO+1 doublet lie within the gap between the occupied and unoccupied states of liquid water.

**Figure 2:**
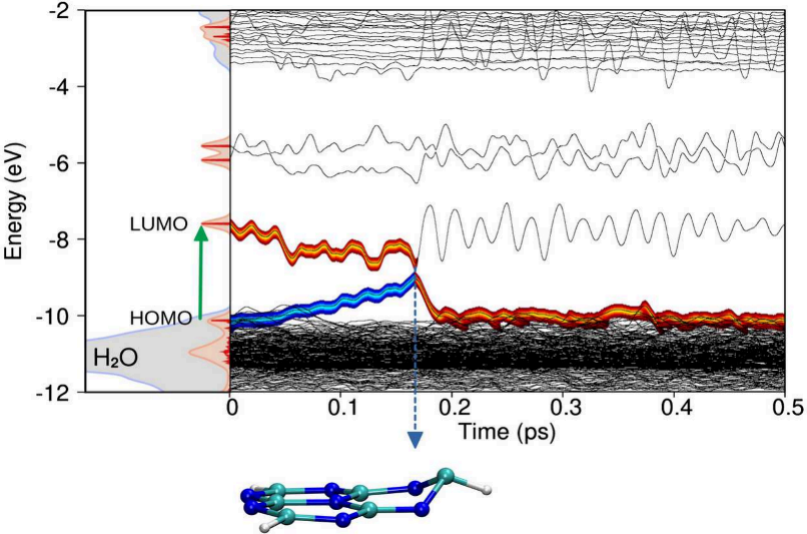
Dynamics of the photoexcited electron–hole pair in solvated HTZ. Left: Electronic density of states (DoS) projected onto quantum H_2_O molecules (gray) and HTZ (red, scaled by 4). Molecular orbitals (MOs) are labeled; the green arrow indicates the S_1_ π → π* transition. Right: Time evolution of MO energies (black lines) from excited-state NAMD. The 1b_1_ band of water spans −10 to −12 eV; conduction states appear between −2 and −4 eV. The HOMO aligns with the 1b_1_ band edge, while the LUMO lies in the water band gap. Thick orange and blue traces represent electronic and hole occupations, respectively; their merging at *t* ≈ 0.17 ps indicates nonradiative recombination. Inset: Distorted HTZ at the conical intersection.

The thick orange and blue traces represent the electron and hole wave function occupations of the molecular orbitals (MOs), respectively. At *t* ≈ 0.17 ps, the blue trace merges with the orange one, signaling nonradiative electron–hole recombination. These traces also indicate the dynamics of the Ehrenfest-CSDM pointer states for the electron and hole wave functions. The HOMO-to-LUMO π → π* excitation induces geometric distortions in HTZ, lowering the LUMO energy while raising the HOMO energy. This evolution leads to the formation of conical intersections, which facilitate nonradiative decay to the ground state at *t* ≈ 0.17 ps. The geometric distortions are primarily associated with puckering deformation modes, as illustrated in the inset of Figure 2. Immediately after relaxation to the electronic ground state, the HOMO and LUMO energies resume oscillations around their equilibrium values. A complementary perspective is provided in Figure S15, which shows the projected density of states (PDOS) for water and HTZ as a function of time.

Four out of ten excited-state trajectories decay to the ground state via this pathway, while the remaining trajectories follow a water-to-HTZ photorelaxation pathway, which we analyze next. The analysis of all ten trajectories resulting from S_1_ → S_0_ photorelaxation is provided in the Supporting Information.

Figure 3a illustrates the electron–hole pair dynamics for a different trajectory of the solvated HTZ system, also initiated by the S_0_ → S_1_ vertical excitation. Energetically, the electron–hole pair dynamics resemble the previous case, culminating in nonradiative recombination at approximately 0.21 ps. However, the net charge evolution of the HTZ molecule, shown in Figure 3b, reveals an electron transfer from water to HTZ during the photorelaxation process. This can also be described as the transfer of the hole wave function from the HOMO of HTZ to the surrounding water molecules. At 0.21 ps, this charge transferred excited state decays via electron–hole recombination through electron back-transfer to the water. The photorelaxation process is schematically depicted in Figure 3c and proceeds through the following steps: (0) photon absorption and (1) the S_1_ π → π* transition, (2) water-to-HTZ electron transfer, and (3) electron–hole recombination via electron back-transfer to the water.

**Figure 3:**
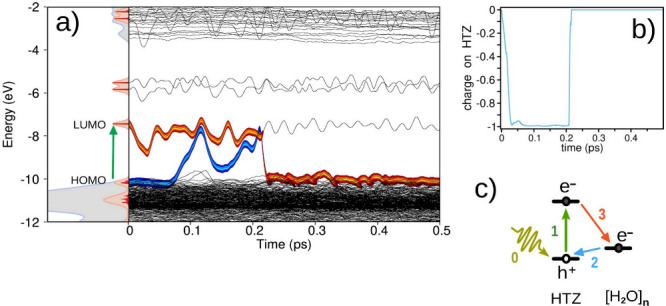
(a) Dynamics of water-to-HTZ electron transfer photorelaxation from the S_1_ state. Left: Electronic density of states (DoS) projected onto quantum H_2_O (gray) and HTZ (red, scaled by 4). Molecular orbitals (MOs) are labeled; the green arrow indicates the S_1_ π → π* transition. Right: Time evolution of MO energies (black lines) from excited-state NAMD. Thick orange and blue traces represent electronic and hole occupations, merging at *t* ≈ 0.21 ps, signaling nonradiative recombination. (b) Net charge on HTZ following S_1_ excitation; negative values indicate water-to-HTZ electron transfer. (c) Schematic of photorelaxation: (0) photon absorption, (1) S_1_ π → π* excitation, (2) electron transfer from water to HTZ, and (3) electron–hole recombination via back-transfer.

Although Figure 3a effectively illustrates the electron–hole pair dynamics, it does not explicitly reveal the nature of the state occupied by the hole (or electronic vacancy), since at *t* = 0, the occupied molecular orbitals (MOs) of water and HTZ are hybridized within the broad 1b_1_ band of liquid water. To clarify this aspect, Figure 4 shows the projected density of states (PDOS) for water and HTZ, computed on the fly during the excited-state dynamics shown in Figure 3.

**Figure 4:**
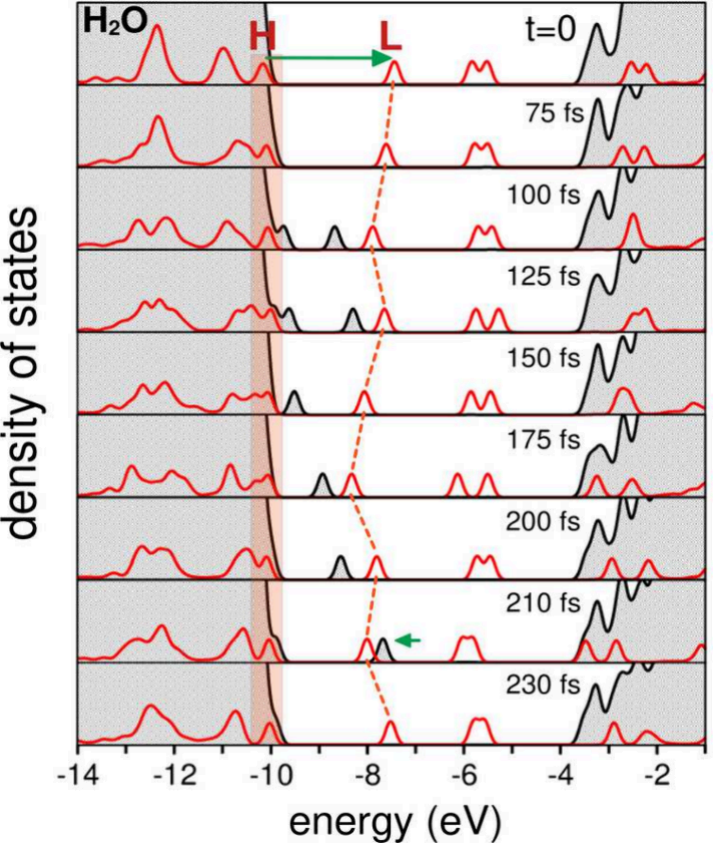
Projected density of states (PDOS) for water (gray) and HTZ (red) over time, illustrating water-to-HTZ electron transfer photorelaxation. Energy levels are shown from *t* = 0 to 230 fs following photoexcitation (horizontal green arrow). The HOMO (H) remains stationary, as indicated by the shaded region. At *t* ≈ 100 fs, an energy level associated with a cationic water cluster detaches from the water valence band (gray peak). Electron–hole recombination via electron back-transfer occurs at *t* ≈ 210 fs, reducing the cationic water state.

Figure 4 presents snapshots of the energy level dynamics over time (*t* = 0 to 230 fs) following the photoexcitation of HTZ, as indicated by the horizontal green arrow. The HOMO (H) and LUMO (L) of HTZ are labeled. The PDOS of water is shown in gray, while the red peaks correspond to HTZ.

A key observation is that the HOMO of HTZ remains fixed in energy throughout the dynamics, as indicated by the shaded rectangular area. In contrast, a discrete energy level associated with a subset of water molecules detaches from the 1b_1_ band of liquid water and shifts into the band gap region. This single quantum state, delocalized over a few positively charged water molecules (as will be shown later), rises above the HOMO of HTZ and becomes occupied by the hole wave function, corresponding to the thick blue trace in Figure 3a.

At approximately 210 fs, the detached quantum state of water becomes resonant with the LUMO of HTZ, facilitating electron–hole recombination. As expected, once the solvated HTZ system returns to the electronic ground state, the shifted quantum state of water vanishes and reintegrates into the 1b_1_ band.

In a separate trajectory, illustrated in Figure 5a, we identify another water-to-HTZ photorelaxation trajectory. As shown in Figure 5c, the electron is transferred from a nearby (H_2_O)_*n*_^+^ cluster to the vacant lone pair orbitals on nitrogen atoms in heptazine. Subsequently, Figure 5d captures the back electron transfer (back-ET) event, wherein the electron returns from HTZ to the water cluster via a trigonal pyramidal carbon atom bearing a partial negative charge and acting as an intermediate carbanion. The associated H–C–N bond angles are approximately 109°, indicative of a pyramidalized, sp^3^-like configuration. This transient geometry supports the formation of a carbanion intermediate,^50^ stabilized by inductive and resonance effects within the HTZ molecule. This interpretation is further supported by Figure 5e, which shows the electron and hole wave functions during the back-ET event at *t* = 590 fs, along with the hemibonded (H_2_O)_2_^+^ cluster. Between the forward and back electron transfer events, Figure 5b reveals the stabilization of the hole within hemibonded (H_2_O)_*n*_^+^ cluster, characterized by short O–O distances (*d*_O–O_ ≲ 2.4 Å) and the absence of conventional hydrogen bonding. Following recombination, the (H_2_O)_*n*_^+^ cluster dissociates, and the constituent water molecules rapidly disperse.

**Figure 5:**
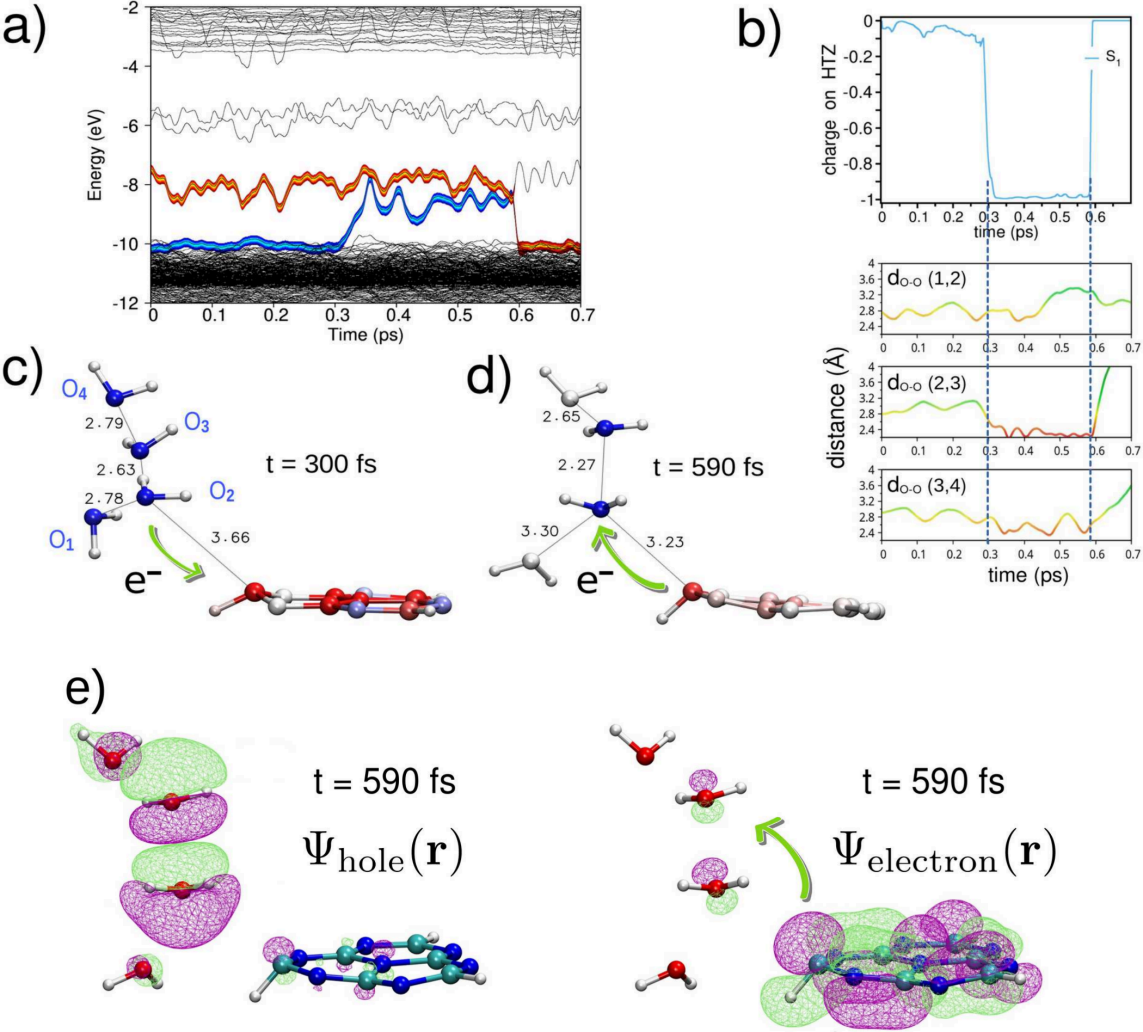
(a) Time evolution of the water-to-HTZ electron transfer following the S_1_ π → π* transition in an excited-state NAMD simulation. (b) Top panel: net charge on HTZ over time for the S_0_ → S_1_ excitation (blue). Negative values indicate electron transfer from water to HTZ. Bottom panel: distance between oxygen atoms (*d*_O–O_), with the color scale indicating short (red) and long (green) distances. (c) Snapshot of the electron transfer (ET) from the (H_2_O)_*n*_^+^ water cluster to the HTZ molecule at *t* = 300 fs. Water molecules are labeled 1 to 4. (d) Same as c) for back-ET event at *t* = 590 fs. (e) Detail of the back-ET event showing hole and electron wave functions.

A detailed analysis of ten photorelaxation trajectories is presented in Figures S11–S20 of the Supporting Information.

It is worth highlighting key differences between the cationic hole and the excess electron in water. As shown earlier, the cationic hole relaxes into an approximately linear chain (2 ≤ *n* ≤ 4) of water molecules that avoid hydrogen bonding. In contrast, the solvated electron, according to the generally accepted model, is characterized by a one-electron wave function localized within a quasi-spherical solvent cavity, typically formed by four to six coordinated water molecules.^51^ Another fundamental difference between these two quasiparticles is their respective lifetimes. The hydrated electron has a lifetime exceeding 100 ps,^52^ whereas the cationic dimer (H_2_O)_2_^+^ exhibits a much shorter lifetime, typically undergoing rapid proton transfer (within sub-100 fs) to a neighboring water molecule, leading to the formation of aqueous H^+^ and a hydroxyl radical (OH^•^).^53–55^

Experimental insights into the elusive cationic hole in water are typically obtained through photoionization experiments. However, direct ionization produces highly energetic, nonequilibrium water cations,^56,57^ complicating the characterization of these transient species.^54^ In contrast, the photorelaxation process described here generates water cations in an almost adiabatic manner, offering a more controlled framework for their study.

Another key insight from these simulations is the critical role of solvation shell size in capturing solvent effects on photorelaxation. To demonstrate this, we repeated simulations (Figures S12 and S16, Supporting Information) using the same initial conditions but reducing the quantum droplet radius to 5 Å (from the central nitrogen of HTZ), corresponding to ∼9 quantum water molecules versus 125 for the 10 Å case. We observe the following changes. The broad 1b_1_ band of water (which serves as the HOMO of the droplet) becomes a narrow, sparse band, preventing full water-to-chromophore electron transfer; at most, we observe a 30% transfer. As a result, the hemibonded cationic complex does not form. Instead, hydrogen-bonded water molecules gain prominence due to their closer proximity.

It is also essential to consider finite-size effects, HTZ solubility, water radical stability, and alternative photorelaxation pathways.

Numerous studies have shown that the valence and conduction band edge states of bulk water correspond to delocalized electronic states.^58–60^ As a result of this delocalization and the associated charge transfer effects, valence excitations in condensed-phase water involve multiple water molecules. For example, in Stoyanov’s model for aqueous H^+^,^61^ the positive charge is delocalized over six water molecules. Consequently, small water clusters often fail to capture the electronic and dynamic complexity of liquid water. In agreement with these findings, our simulations highlight the necessity of including a sufficiently large quantum mechanical droplet to accurately model the behavior of solvated systems. We observed that finite-size effects become significant as the droplet radius decreases below 10 Å, underscoring the need for extended solvation shells to reliably describe photorelaxation dynamics in water. However, increasing the size of the QM water droplet leads to an exponential rise in computational cost, making extensions beyond 10 Å impractical for our method. Notably, tests with a slightly smaller droplet (*R* = 9 Å) showed no change in the occurrence or characteristics of the water-to-chromophore first electron transfer event.

Due to its relevance as a photocatalyst for hydrogen production,^16^ the photophysics of the heptazine–water complex has been the focus of several computational investigations.^7,9,18^ These studies employed first-principles methods to explore reaction pathways in HTZ–water clusters that may lead to water photolysis. According to their findings, the overall photocatalytic cycle that generates the H_3_O radical involves two photoinduced steps mediated by the HTZ chromophore. In the first step, a proton-coupled electron transfer (PCET) reaction abstracts a hydrogen atom from a water molecule hydrogen-bonded to a peripheral nitrogen atom of HTZ, yielding heptazinyl (HTZ–H) and a hydroxyl radical (OH^•^).^9,18^ In the second step, driven by the absorption of a second photon, an H_3_O radical forms via hydrogen transfer from HTZ–H to a neighboring water molecule, thereby regenerating the HTZ chromophore.

The experimental formation of photoinduced heptazinyl radicals was recently demonstrated using a carbon-nitride monomer hydrogen-bonded to methoxyphenol.^62^ However, heptazine’s low solubility in most solvents,^63,64^ especially water, hinders direct experimental study. Derivatives with improved solubility are often used, but their bulky substituents disrupt water–heptazine interactions, complicating efforts to clarify photocatalytic mechanisms in the unmodified system.

The reaction mechanism proposed here for production of H_3_O^+^ cation differs from the aforementioned model in two key aspects: it requires only a single photon absorption by heptazine and does not involve a hydrogen-bonded water molecule or a PCET mechanism for hydrogen abstraction. Instead, our simulations reveal that photoexcitation triggers electron transfer from water to HTZ, creating a cationic hole that is stabilized by a (H_2_O)_*n*_^+^ cluster.

The molecular mechanics force field employed in our study, based on the flexible TIP3P model,^46,47^ does not account for the decay of the cationic water cluster into basic water species. Nonetheless, this process is known to occur with high efficiency under experimental conditions, typically within 100 fs.^53,65–67^ Water ionization also yields various metastable radicals.^68^ Schaefer et al. identified 14 stationary points for the (H_2_O)_2_^+^ radical cation using coupled-cluster theory,^54^ with the proton transferred form (H_3_O^+^···OH) as the most stable. The second-lowest energy configuration, the hemibonded (H_2_O)_2_^+^ structure, is metastable and separated from the global minimum by an energy barrier of 8 kcal/mol.^54,55^ Thus, although our simulations do not explicitly capture the formation of H_3_O^+^ and other water radicals, these species are expected to form readily upon S_0_ → S_1_ excitation of heptazine.

We cannot entirely rule out H-bond-assisted relaxation, as our water force field is nondissociative and therefore cannot capture PCET processes. Conversely, the HTZ–water cluster models used in previous studies lack the extended hydrogen-bond network and dynamic solvent environment of liquid water, and thus cannot describe the solvation-driven mechanism observed in our work. Future studies employing reactive water models that support PCET in a dynamic solvent environment may help reconcile these two perspectives.

In conclusion, we report a previously unrecognized photorelaxation pathway in which initial electron transfer (ET) occurs via direct hybridization between the 1b_1_ lone pair p-orbitals of water and lone pair orbitals on heptazine nitrogen atoms, without hydrogen-bond mediation. The subsequent back-ET proceeds through a pyramidalized aromatic carbon acting as a carbanion intermediate, enabling electron–hole recombination.

This water-to-HTZ ET mechanism forms a transient cationic water cluster, (H_2_O)_*n*_^+^ (2 ≤ *n* ≤ 4), stabilized in a hemibonded configuration. This intermediate recombines with the excess electron on HTZ within subpicoseconds. Under experimental conditions, this cluster is expected to decay rapidly,^53,65–67^ producing aqueous H_3_O^+^.

Our simulations underscore the role of solvent fluctuations and nuclear motion in shaping photorelaxation. Finite-size effects significantly impact excited-state dynamics, highlighting the need for large quantum droplets to capture the delocalization and charge transfer behavior of bulk water.

These findings suggest that reactive water radical photogeneration in the heptazine photocatalytic cycle may proceed via mechanisms beyond the traditional proton-coupled electron transfer (PCET). While prior studies proposed a two-photon process,^7,9,18^ our results indicate that single-photon excitation suffices to drive water-to-HTZ ET, forming hydroxyl and hydronium radicals via rapid proton transfer within water.

Future studies with explicit treatment of radical formation dynamics are underway to refine our understanding of these processes and their implications for solar energy conversion and photocatalysis.

## Supplementary Material







## Notes

(1) Theoretical methods, (2) molecular mechanics force field parameters, (3) extended Hückel model and parameters, (4) excited-state dynamics trajectories (PDF)
Photorelaxation process (AVI)
Photorelaxation process (AVI)
