# Supplementary material for: Photorelaxation via Water-Mediated Electron Transfer in Fully Solvated Heptazine
Source: J Phys Chem Lett. 2025 Jul 31;16(32):8075–83. doi: 10.1021/acs.jpclett.5c01896 (PMC12359197; doi:10.1021/acs.jpclett.5c01896)
Supplement: Supplementary file 1 [file jz5c01896_si_001.pdf]

Supporting Information for: Photorelaxation via Water-Mediated Electron Transfer in Fully Solvated Heptazine

Laure de Thieulloy,[†] Robson S. Oliboni,[‡] Piotr de Silva,[†] and Luis G. C. Rego[*,¶]

†*Department of Energy Conversion and Storage, Technical University of Denmark, 2800 Kongens Lyngby, Denmark*

‡*Department of Chemistry, Universidade Federal de Pelotas, Pelotas, RS, 96010-900, Brazil*

¶*Department of Physics, Universidade Federal de Santa Catarina, SC, 88040-900, Brazil*

E-mail: luis.guilherme@ufsc.br

1 Theoretical Model

The Dynemol quantum–classical method for excited-state nonadiabatic dynamics, using Coherent Switches with Decay of Mixing (CSDM),[1–3] is briefly described below. More details are provided in earlier studies.[4–6]

The time-dependent Schrödinger equation (TDSE) is solved for the electronic degrees of freedom

$$i\hbar\frac{\partial}{\partial t}\left|\Psi(\mathbf{r};t)\right\rangle = \hat{H}_{el}(\mathbf{R}_t)\left|\Psi(\mathbf{r};t)\right\rangle \ , \tag{1}$$

where $\mathbf{r}$ designates the electronic coordinates, $\mathbf{R}_t \equiv \mathbf{R}(t)$ are the time-dependent nuclear coordinates and $\hat{H}_{el}(\mathbf{R}_t)$ is the time-dependent extended Hückel hamiltonian for an instantaneous molecular configuration. We use the Generalized Amber Force Field (GAFF2, version 2.2.20) to describe the nuclear dynamics

$$\begin{aligned} V_{GS}^{MM}(\{\mathbf{R}\}) &= \sum_{\text{bonds}} K_b(\mathbf{R}-\mathbf{R}_0)^2 + \sum_{\text{angles}} K_\theta(\theta-\theta_0)^2 + \sum_{\text{dihedrals}} V_n(1+cos(n\phi-\gamma)^n \\ &+ \sum_{i<j} 4\varepsilon_{ij}\left[\left(\frac{\sigma_{ij}}{\mathbf{R}_{ij}}\right)^{12} - \left(\frac{\sigma_{ij}}{\mathbf{R}_{ij}}\right)^{6}\right] + \sum_{i<j}\frac{q_j q_i}{4\pi\epsilon_0\mathbf{R}_{ij}} \ , \end{aligned} \tag{2}$$

where $\mathbf{R}$ is the atomic position and $\mathbf{R}_{ij} = |\mathbf{R}_i - \mathbf{R}_j|$ is the distance between atoms i and j. Likewise, θ and ϕ are the angular and dihedral torsional variables. The parameters $\mathbf{R}_0$ and θ_0 are the equilibrium bond length and angle; K_b, K_θ and V_n (where $V_n = \mathtt{v}_n/2$, as it appears in some documentation[7]) are the intramolecular FF parameters, and ε_{ij} and σ_{ij} are the Lennard-Jones parameters. The FF parameters are presented in Section 2 of this document.

To perform the nonadiabatic nuclear dynamics, we extend the ground-state molecular mechanics (MM) framework to the excited-state, where the nuclear dynamics are governed

by the classical equations of motion

$$\dot{\mathbf{R}} = \mathbf{P}/M \; , \tag{3}$$

$$\dot{\mathbf{P}} = -\nabla_{\mathbf{R}} \langle \Psi(\mathbf{r};t)|V(\mathbf{r},\mathbf{R})|\Psi(\mathbf{r};t)\rangle_{\mathbf{r}} \; , \tag{4}$$

where we approximate the potential energy term as follows:

$$\langle \Psi(\mathbf{r};\mathbf{R},t)|V(\mathbf{r},\mathbf{R})|\Psi(\mathbf{r};\mathbf{R},t)\rangle_{\mathbf{r}} \approx V_{GS}^{MM}(\mathbf{R}) + V_{EH}\left[\Psi^{el}(\mathbf{R},t), \Psi^{hl}(\mathbf{R},t)\right] \; . \tag{5}$$

In this expression, the excited-state interatomic potential is decomposed into the ground-state force field potential, V_{GS}^{MM}, and an excitation-induced correction term, $V_{EH}\left[\Psi^{el}, \Psi^{hl}\right]$. The rationale behind this approximation is that the classical force field V_{GS}^{MM} accurately captures the forces arising from the occupied orbitals. The term V_{EH}, a functional of the photoexcited electron and hole wavefunctions, is introduced to account for the excited-state charge redistribution. It gives rise to nonadiabatic Hellmann–Feynman–Pulay forces, which serve as excitation-induced corrections to the ground-state potential, effectively capturing the back-reaction of the evolving electronic structure on the nuclear dynamics. This approach allows us to retain fixed ground-state charges while still incorporating essential excited-state effects, and it ensures conservation of the total energy of the system (classical + quantum) throughout the dynamics. The term V_{EH} is calculated on-the-fly as

$$V_{EH}\left[\Psi^{el}(\mathbf{R},t), \Psi^{hl}(\mathbf{R},t)\right] = Tr\left[\rho^{EH}(\mathbf{R},t)\mathbf{H}(\mathbf{R}_t)\right] \tag{6}$$

where $\rho^{EH} = |\Psi^{el}\rangle\langle\Psi^{el}| - |\Psi^{hl}\rangle\langle\Psi^{hl}|$ is the electron-hole density matrix. In the excited-state, the force produced on atom N by an e-h excitation is given by

$$\mathbf{F}_N = -\nabla_N V_{EH}\left[\Psi^{el}(\mathbf{R},t), \Psi^{hl}(\mathbf{R},t)\right] \; , \tag{7}$$

where $\nabla_N \equiv \nabla_{\mathbf{R}_N}$. This force is responsible for the electronic back-reaction on the classical degrees of freedom and it gives rise to nonadiabatic nuclear dynamics effects.[4,5,8] In this approach, the total energy of the excited system consists of the overall classical energy of the nuclei (kinetic plus potential) as given by the molecular mechanics (MM) formalism, along with the quantum energy of the excited electron-hole pair, given by $E_{QM} = Tr\left[\rho^{EH}\mathbf{H}\right]$.

In our dynamics method, the electron and hole are described by distinct wavefunctions: the electron propagates in the excited-state manifold, and the hole in the valence manifold. At the conical intersection, the Ehrenfest force acting on the nuclei arises from both wavefunctions, as does the nonadiabatic coupling vector.[5] We believe this framework provides a minimal yet effective multi-reference character for describing the conical intersection region.

The implementation details of the CSDM method within the semiempirical framework is provided elsewhere.[6] All semiempirical calculations were conducted using the Dynemol simulation package.[9]

2 Molecular Mechanics Force Field Parameters

2.1 Structures

Figure S1 shows an excellent match between the geometries optimized by the classical force field based on the Generalized Amber Force Field[10] and those obtained from DFT for the heptazine molecule, as well as for the monomer, dimer, and trimer of the melem (MLM) compound.

In addition to accurately reproducing the overall geometry, the classical force field correctly describes the two lowest-energy isomeric configurations of the melem trimer, with an energy deviation of approximately 2.5 kcal/mol relative to the DFT wB97xD/6-31g(d,p) calculations, as shown in Figure S2.

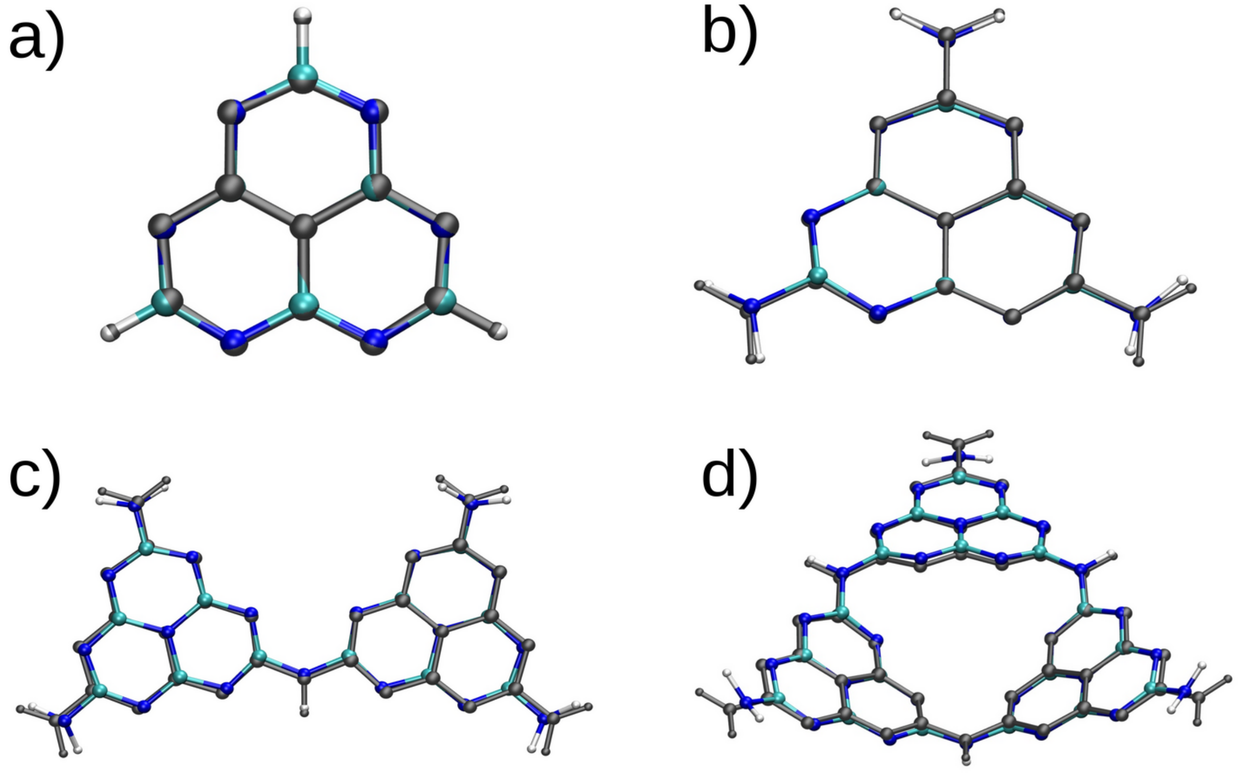

Figure S1: Comparison of optimized molecular structures based on ground-state energy obtained using the molecular mechanics framework (blue, cyan, and white atoms) and the DFT method (solid color) with the long-range corrected hybrid functional wB97xD and the 6-31g(d,p) basis set. Molecular structures shown are: (a) heptazine (HTZ) molecule, (b) melem (MLM) molecule, (c) melem dimer, and (d) melem trimer.

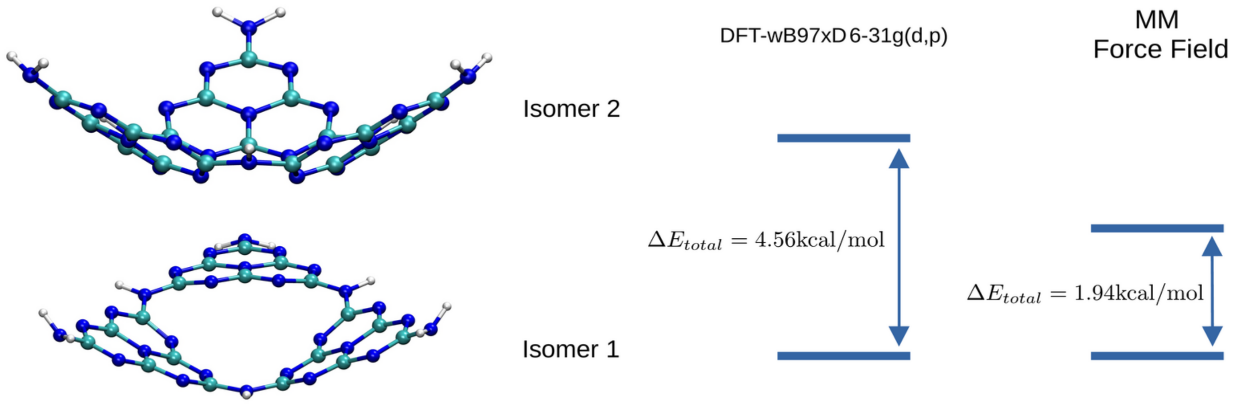

Figure S2: Lowest-energy isomers of the melem trimer structure, with corresponding total energies calculated using the DFT and MM frameworks.

2.2 Force Field Parameters

2.2.1 Heptazine-Water Model

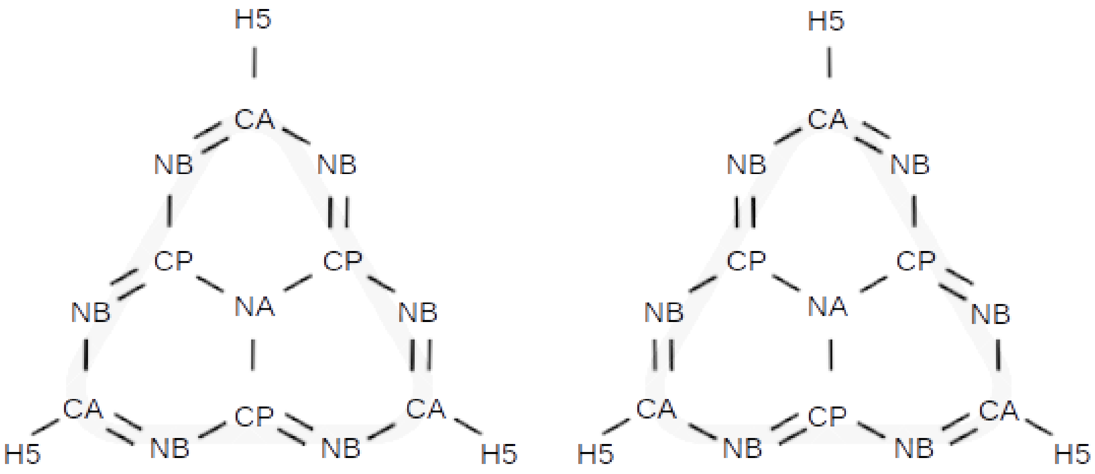

Figure S3: Atom types for the heptazine molecule, as defined by the GAFF2 force field model.[10] The gray contour highlights the external aromatic ring.

Table S1: Force field parameters as defined by the GAFF2 (version 2.2.20) model[10] for the heptazine molecule, along with flexible TIP3P parameters for the flexible water molecule's OW and HW atoms.[11,12]

Bond/Angle	Energy (kcal·mol^{-1}/Å^2)	Length (Å)	Angle (degrees)	Energy (kcal·mol^{-1})
CP-NA	296.53	1.4172	-	-
CP-NB	388.52	1.3377	-	-
CA-NB	386.49	1.3392	-	-
CA-H5	357.59	1.0880	-	-
OW-HW	450.00	0.9572	-	-
CA-NB-CP	-	-	118.09	82.90
H5-CA-NB	-	-	115.79	50.16
NB-CP-NB	-	-	125.84	73.26
NB-CA-NB	-	-	127.30	72.76
CP-NA-CP	-	-	118.01	78.32
NA-CP-NB	-	-	114.96	74.32
HW-OW-HW	-	-	55.00	104.52

Table S2: Dihedral parameters as defined by the GAFF2 (version 2.2.20) model[10] for the heptazine (HTZ) molecule. Improper Dihedral: central atom in 3rd position

Torsion Dihedral	N. of Paths	Energy (kcal/mol)	Phase (degrees)	Periodicity	Notes
X-CA-NB-X	2	9.60	180	2	same as X-CA-NC-X
X-CP-NA-X	4	9.30	180	2	from parm99 (X-CR-NA-X)
X-CP-NB-X	2	9.60	180	2	from GAFF2 (X-CA-NB-X)
	2	10.0	180	2	similar to parm99 (X-CR-NB-X)
**Improper Dihedral**		**Energy (kcal/mol)**	**Phase (degrees)**	**Periodicity**	**Notes**
NB-NB-CA-H5	-	1.10	180	2	based on C6H6 nmodes
CP-CP-NA-CP	-	1.10	180	2	from GAFF2 (CA-CA-NA-C3)
NA-NB-CP-NB	-	1.10	180	2	GAFF2 (NA-N2-CA-N2)<br>parm99 (N2-NA-CA-NC)

Table S3: AM1-BCC charges and Lennard-Jones parameters for the HTZ molecule, along with the flexible TIP3P parameters for the flexible H_2O molecule's OW and HW atoms.[11,12]

Atom Type	Charge	ε (kcal/mol)	σ (Å)
NA	-0.538312	0.170	3.648
CP	0.839687	0.086	3.816
NB	-0.736812	0.170	3.648
CA	0.735087	0.086	3.816
H5	0.078287	0.015	2.718
OW	-0.83400	0.1521	3.5364
HW	0.41700	0.046	0.449

2.3 HTZ-Water System Thermalization

For equilibration, molecular dynamics (MD) simulations were performed with the Gromacs 2024.2 software.[13] The system was thermalized at the NVT ensemble for 500 ps, followed by a NPT simulation of 2 ns. The Berendsen thermostat (T = 298 K, $\tau_T = 1.0$ ps) and barostat (P = 1 atm, $\tau_P = 0.8$ ps) were used for temperature and pressure coupling. The particle-mesh Ewald (PME) and the Switch algorithm were used to treat electrostatic and van der Waals interactions, respectively. Energy and pressure dispersion corrections were used throughout the simulations.

2.4 Radial Distribution Functions of the HTZ-Water System

Figure S4 shows the radial distribution functions (RDFs) for liquid water in the HTZ-WAT simulation box, modeled using the modified (flexible) TIP3P potential.[11,12] The intermolecular peaks are labeled by their radial distances. The results exhibit good agreement with data from the literature.[12] Notably, the presence of the HTZ molecule has a negligible impact on the RDFs $g_{\text{OW-OW}}$, $g_{\text{HW-HW}}$, and $g_{\text{OW-HW}}$, indicating that the overall hydrogen-bonding structure of water remains largely unperturbed.

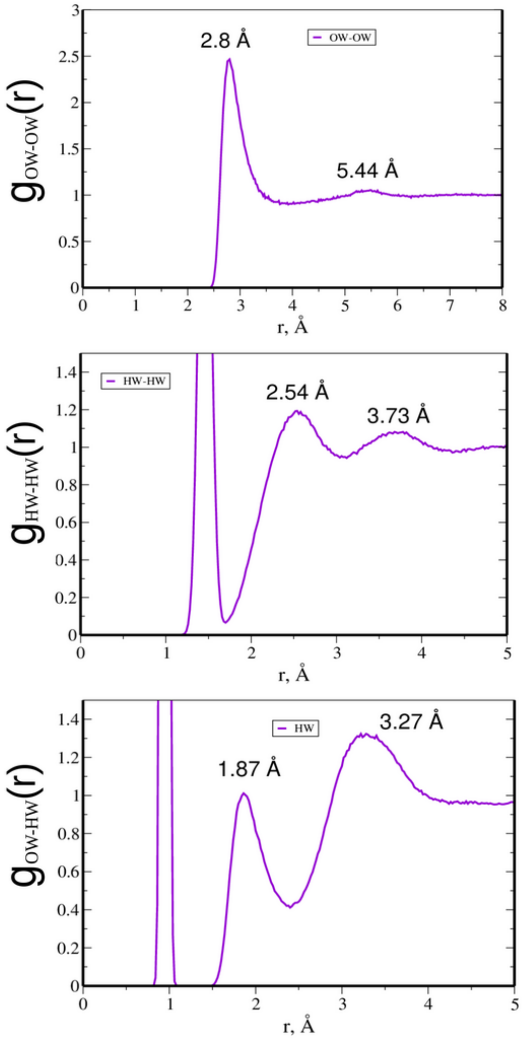

Figure S4: Radial distribution functions (RDFs) for liquid water in the HTZ-WAT simulation box using the modified TIP3P water model. From top to bottom: oxygen–oxygen $g_{\mathrm{OW-OW}}$, hydrogen–hydrogen $g_{\mathrm{HW-HW}}$, and oxygen–hydrogen $g_{\mathrm{OW-HW}}$ RDFs.

3 Extended-Hückel Model and Parameters

3.1 Extended-Hückel Model

Here we examine the long range interaction between the solute and a solvent molecule within the framework of the extended-Hückel tight-binding model. To establish the notation, we define Slater-type atomic orbitals (STOs) as:

$$\langle \mathbf{r}|i;\mathbf{R}_I\rangle \equiv \phi_i(\mathbf{r}-\mathbf{R}_I) = \sqrt{\frac{1}{(2n)!}}(2\zeta)^{n+1/2}(\mathbf{r}-\mathbf{R}_I)^{n-1}\exp\Big[-\zeta(\mathbf{r}-\mathbf{R}_I)\Big]Y_{lm}(\theta,\phi)\ , \tag{8}$$

where $Y_{lm}(\theta,\phi)$ are spherical harmonics, and $i=\{n,l,m\}$ denotes the quantum numbers associated with the orbital. The parameter ζ is a semiempirical quantity that characterizes the spatial extent of the orbital.

In solute-solvent simulations, the Hamiltonian matrix element consists of two contributions: a short-range (SR) term based on the extended-Hückel model and a long-range (LR) term arising from the electrostatic potential.

The short-range Hamiltonian is based on the modified extended-Hückel formalism:

$$\langle\phi_i|H^{SR}|\phi_j\rangle = \chi_{ij}S_{ij}\ , \tag{9}$$

where S_{ij} is the overlap between pairs of Slater-type orbitals, $\chi_{ii}=V_i+V_i^{\text{shift}}$ (for $i=j$) and

$$\chi_{ij} = \left[\frac{\kappa_{ij}}{2}\left(V_i+V_j\right)+\frac{1}{2}\left(V_i^{\text{shift}}+V_j^{\text{shift}}\right)\right]\ , \tag{10}$$

where $\overline{K}_{ij}=\frac{1}{2}(K_i+K_j)$ and

$$\kappa_{ij} = \overline{K}_{ij}+\Delta^2+\Delta^4(1-\overline{K}_{ij}) \tag{11}$$

is the modified Wolfsberg-Helmholz formula proposed by Hoffmann to prevent unphysical

orbital mixing.[14] The parameter Δ is given by

$$\Delta = \frac{V_i - V_j}{V_i + V_j}. \tag{12}$$

The parameters used to construct the Hamiltonian matrix elements in Equation (9) are provided in Section 3.2.

Additionally, the semiempirical parameter V_{shift} is introduced to align the highest occupied molecular orbital (HOMO) energies of independent molecular systems while maintaining their internal molecular orbital (MO) energy spacing. The matrix element $\langle \phi_i | H^{SR} | \phi_j \rangle$ is evaluated for all pairs of Slater-type orbitals (STOs), $\{i, j\}$, within a cutoff radius of 12 Å, irrespective of whether the STOs belong to the same molecule or different molecules. As such, it accounts for both intramolecular and intermolecular (through-space) electron-transfer effects.

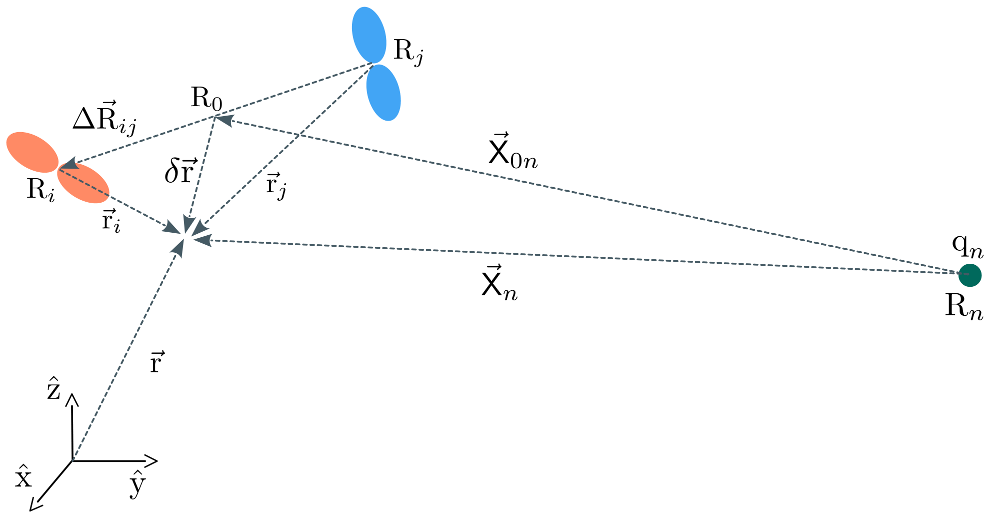

Figure S5: Schematic representation of the long-range intermolecular electrostatic interaction. Slater-type orbitals (STOs) are centered at atomic positions $\mathbf{R}_i$ and $\mathbf{R}_j$, while the long-range charge center q_n is located at $\mathbf{R}_n$. The centroid of the orbital centers is given by $\vec{R}_0 = \left(\vec{R}_i + \vec{R}_j\right)/2$, and the relative coordinate between them is defined as $\Delta\vec{R}_{ij} = \vec{R}_i - \vec{R}_j$.

Now, we derive an approximation for the matrix element of the electrostatic interaction. Consider two independent atomic centers, denoted by $\mathbf{R}_i$ and $\mathbf{R}_j$ (see Figure S5). The

electrostatic potential at a nearby point $\mathbf{r}$, generated by a localized charge distribution q_n at $\mathbf{R}_n$, can be approximated using a multipole expansion. The matrix element of the electrostatic interaction between the STOs $|i;\mathbf{R}_I\rangle$ and $|j;\mathbf{R}_J\rangle$ is then given by:

$$\begin{aligned} \langle i;\mathbf{R}_I|\, \frac{q_n}{\mathsf{x}_n}\, |j;\mathbf{R}_J\rangle &\approx \langle\phi_i|\, q_n\, \left\{\frac{1}{\mathsf{x}_{0n}} - \frac{\vec{\mathsf{x}}_{0n}}{|\vec{\mathsf{x}}_{0n}|^3}\cdot\delta\vec{r}\right\}|\phi_j\rangle & (13)\\ &\approx \langle\phi_i|\, q_n\, \left\{\frac{1}{\mathsf{x}_{0n}} - \frac{\vec{\mathsf{x}}_{0n}}{|\vec{\mathsf{x}}_{0n}|^3}\cdot\left(\vec{r}-\vec{R}_0\right)\right\}|\phi_j\rangle\ , & (14)\end{aligned}$$

where the molecular mechanics force field charge is used for q_n and $\vec{R}_0 = \left(\vec{R}_i + \vec{R}_j\right)/2$ represents the centroid of orbital centers $\mathbf{R}_i$ and $\mathbf{R}_j$.

The expression in Eq. (14) can be rearranged to yield

$$\begin{aligned} \langle\phi_i|\,\frac{q_n}{\mathsf{x}_n}\,|\phi_j\rangle &\approx \frac{q_n}{\mathsf{x}_{0n}}S_{ij} - q_n\frac{\vec{\mathsf{x}}_{0n}}{|\vec{\mathsf{x}}_{0n}|^3}\cdot\langle\phi_i|\left(\vec{r}-\vec{R}_0\right)|\phi_j\rangle & (15)\\ &\approx \frac{q_n}{\mathsf{x}_{0n}}S_{ij} - q_n\frac{\vec{\mathsf{x}}_{0n}}{|\vec{\mathsf{x}}_{0n}|^3}\cdot\left(\langle\phi_i|\vec{r}|\phi_j\rangle - \vec{R}_0 S_{ij}\right)\ , & (16)\end{aligned}$$

where $S_{ij} = \langle\phi_i|\phi_j\rangle$ is an element of the overlap matrix between STO orbitals.

Expression (16) is origin-dependent due to the terms $\langle\phi_i|\vec{r}|\phi_j\rangle$ and $\mathbf{R}_0$. To eliminate this dependence, we express the dipole moment matrix element in terms of local coordinates. Defining:

$$\begin{aligned} \vec{r}_i &= \vec{r} - \vec{R}_i \quad\Longrightarrow\quad \vec{r} = \vec{r}_i + \vec{R}_i & (17)\\ \vec{r}_j &= \vec{r} - \vec{R}_j & (18)\\ &= \left(\vec{r}-\vec{R}_i\right) - \vec{R}_j & (19)\\ &= \vec{r}_i + \Delta\vec{R}_{ij}\ , & (20)\end{aligned}$$

where $\Delta\vec{R}_{ij} = \vec{R}_i - \vec{R}_j$ represents the relative coordinate between atomic centers $\mathbf{R}_i$ and $\mathbf{R}_j$.

$$\begin{aligned}
\langle\phi_i|\vec{r}|\phi_j\rangle &= \int \phi_i(\vec{r}_i)\left[\vec{r}_i + \vec{R}_i\right]\phi_j(\vec{r}_i + \Delta\vec{R}_{ij})\ d^3\vec{r}_i && (21)\\
&= \int \phi_i(\vec{r}_i)\ \vec{r}_i\ \phi_j(\vec{r}_i + \Delta\vec{R}_{ij})\ d^3\vec{r}_i + \vec{R}_i \int \phi_i(\vec{r}_i)\phi_j(\vec{r}_i + \Delta\vec{R}_{ij})\ d^3\vec{r}_i && (22)\\
&= \vec{d}_{ij} + \vec{R}_i S_{ij}\ , && (23)
\end{aligned}$$

where $\vec{d}_{ij}$ represents the dipole integral in the local coordinate system and S_{ij} is the overlap integral.

Substituting Eq. (23) into Eq. (16), we obtain

$$\begin{aligned}
\langle\phi_i|\ \frac{q_n}{\mathsf{x}_n}\ |\phi_j\rangle &\approx \frac{q_n}{\mathsf{x}_{0n}} S_{ij} - q_n \frac{\vec{\mathsf{x}}_{0n}}{|\vec{\mathsf{x}}_{0n}|^3} \cdot \left(\vec{d}_{ij} + \vec{R}_i S_{ij} - \vec{R}_0 S_{ij}\right) && (24)\\
&\approx \frac{q_n}{\mathsf{x}_{0n}} S_{ij} - q_n \frac{\vec{\mathsf{x}}_{0n}}{|\vec{\mathsf{x}}_{0n}|^3} \cdot \left(\vec{d}_{ij} + \frac{\Delta\vec{R}_{ij}}{2} S_{ij}\right) && (25)\\
&\approx \frac{q_n}{\mathsf{x}_{0n}} \left[S_{ij} - \frac{\vec{\mathsf{x}}_{0n}}{|\vec{\mathsf{x}}_{0n}|^2} \cdot \left(\vec{d}_{ij} + \frac{\Delta\vec{R}_{ij}}{2} S_{ij}\right)\right]\ . && (26)
\end{aligned}$$

The final expression is origin-independent and incorporates both monopole and dipole interaction terms. The matrix element for the long-range electrostatic interaction is then given by

$$\langle\phi_i|H^{LR}|\phi_j\rangle = \sum_n{}' \frac{q_n}{\mathsf{x}_{0n}} \left[S_{ij} - \frac{\vec{\mathsf{x}}_{0n}}{|\vec{\mathsf{x}}_{0n}|^2} \cdot \left(\vec{d}_{ij} + \frac{\Delta\vec{R}_{ij}}{2} S_{ij}\right)\right]\ , \qquad (27)$$

where the summation $\sum_n'$ runs over all charge centers q_n located outside a *hardcore exclusion radius* of 3 Å from the centroid $\mathbf{R}_0$.

The long-range matrix element $\langle\phi_i|H^{LR}|\phi_j\rangle$ also couples Slater-type orbitals (STOs), $\{i, j\}$, from different molecular systems that exhibit significant wavefunction overlap ($|S_{ij}| > 10^{-7}$).

3.2 Heptazine Molecule

The extended Hückel (e-Hückel) model Hamiltonian is defined by Eq. (9). For the $C_6N_7H_3$ molecule, we used STOs: 2s and 2p for Carbon and Nitrogen, and 1s for Hydrogen. The corresponding parameters are shown in Table S4. The diagonal elements V_i are associated to the valence-state ionization potentials of each atomic species. The parameter V_{shift} is an energy offset introduced to align the HOMO levels of different molecular species—specifically, the HTZ and water molecules in our case. The parameters K_i are tunable constants that enter Eq. (11) to define the coupling matrix elements κ_{ij}, while S_{ij} denotes the corresponding overlap matrix element between the Slater-type orbitals (STOs).

Table S4: Optimized extended Hückel parameters for the heptazine molecule.

Symbol	EHsymbol	residue	NoAt	Nvalen	n	spdf	IP (V_i)	zeta	K_i	V_{shift}
N	NA	HTZ	7	5	2	s	-25.5357	2.19931	1.74649	1.90
N	NA	HTZ	7	5	2	p	-13.6847	2.19901	1.23317	1.90
C	CP	HTZ	6	4	2	s	-20.8081	2.03912	1.66533	1.90
C	CP	HTZ	6	4	2	p	-10.7387	2.21389	2.95000	1.90
N	NB	HTZ	7	5	2	s	-25.8491	1.87292	1.28684	1.90
N	NB	HTZ	7	5	2	p	-12.8282	1.80741	2.08083	1.90
C	CA	HTZ	6	4	2	s	-20.5613	2.30167	1.57998	1.90
C	CA	HTZ	6	4	2	p	-10.7507	2.23085	3.83360	1.90
H	H5	HTZ	1	1	1	s	-13.6000	1.30000	1.75000	1.90

To parametrize the extended Hückel model Hamiltonian for heptazine, we employed our Adaptive Genetic Algorithm with Symmetry Descriptors, calibrated to reproduce both the charge distribution of the frontier molecular orbitals (MOs) and their excitation energies, as computed using the single-reference algebraic diagrammatic construction scheme to second order (ADC(2)), with the cc-pVDZ basis set in TURBOMOLE.[15]

We selected the ADC(2) method for its balance of stability, accuracy, and computational efficiency, as demonstrated by Plasser and collaborators,[16] who showed that ADC(2) yields consistent results and strong performance in describing the nonradiative decay of the 9H-adenine molecule. ADC(2) is particularly well-suited for modeling dynamical processes within the excited-state manifold. However, caution is warranted in the immediate vicinity of electronic state crossings, where its single-reference character may present limi-

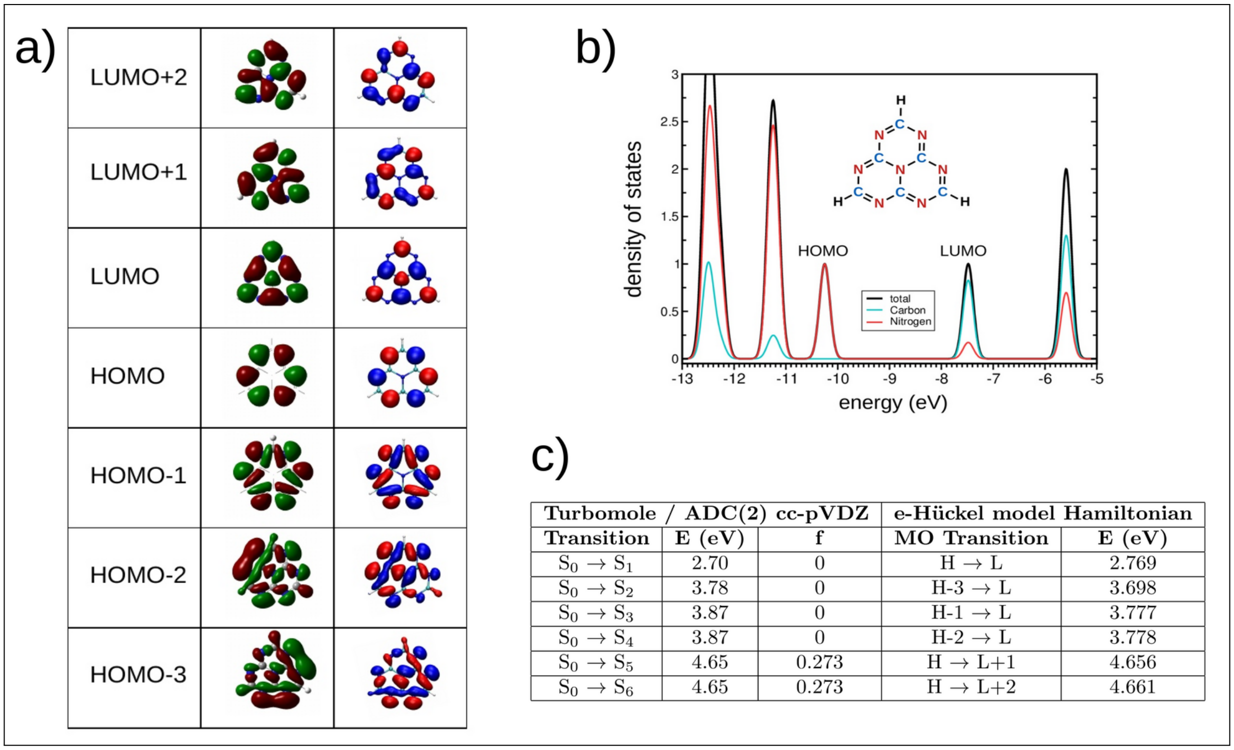

Turbomole / ADC(2) cc-pVDZ			e-Hückel model Hamiltonian	
**Transition**	**E (eV)**	**f**	**MO Transition**	**E (eV)**
$S_0 \rightarrow S_1$	2.70	0	H → L	2.769
$S_0 \rightarrow S_2$	3.78	0	H-3 → L	3.698
$S_0 \rightarrow S_3$	3.87	0	H-1 → L	3.777
$S_0 \rightarrow S_4$	3.87	0	H-2 → L	3.778
$S_0 \rightarrow S_5$	4.65	0.273	H → L+1	4.656
$S_0 \rightarrow S_6$	4.65	0.273	H → L+2	4.661

Figure S6: (a) Isosurfaces of the frontier molecular orbital wavefunctions for HTZ: Kohn-Sham MOs obtained using the DFT-wB97xD/6-31g(d,p) method (green-brown) and the parametrized e-Hückel model Hamiltonian (blue-red). (b) Electronic density of states (DOS) of the HTZ molecule: total DOS (black), projected onto carbon atoms (blue), and projected onto nitrogen atoms (red). (c) Electronic transitions with corresponding excitation energies and oscillator strengths (f) for the $C_6N_7H_3$ molecule in the optimized ground-state geometry.

tations. Nonetheless, ADC(2) remains capable of capturing the key puckering deformations responsible for the nonradiative decay pathways of photoexcited 9H-adenine, whose chemical structure closely resembles that of the HTZ molecule.

It is nonetheless worth noting that more accurate multi-reference methods could also be employed for the parametrization of the semiempirical Hamiltonian, as the energetic landscape remains the primary factor in the parametrization process.

Figure S6-a compares the isosurfaces of the frontier MOs obtained from DFT-wB97xD/6-31G(d,p) (green–brown) and from the parametrized extended Hückel model (blue–red). Figure S6-c compares the corresponding excitation energies computed with ADC(2)/cc-pVDZ in TURBOMOLE with those of the semiempirical model. We emphasize that the parametrization of the extended Hückel Hamiltonian was based on excitation energies computed using

the ADC(2) method, rather than on bare Kohn–Sham orbital energies. As the nonadiabatic molecular dynamics simulations were restricted to the singlet subspace, triplet states were not included in the parametrization.

For the NAMD simulations, it is equally important to assess the quality of the extended Hückel Hamiltonian parametrization as a function of the nuclear coordinates, particularly in the vicinity of electronic state crossings. To this end, Figure S7-a shows the semiempirical excitation energy,

$$V_{EH}\left[\Psi^{el}(\mathbf{R},t),\Psi^{hl}(\mathbf{R},t)\right]=\mathrm{Tr}\left[\rho^{EH}(\mathbf{R},t)\mathbf{H}(\mathbf{R}_t)\right], \tag{28}$$

as defined in Equation (6), for the S_1 state of the heptazine molecule along the adiabatic relaxation pathway. For comparison, excitation energies computed using the ADC(2) method are also shown for selected geometries along the trajectory.

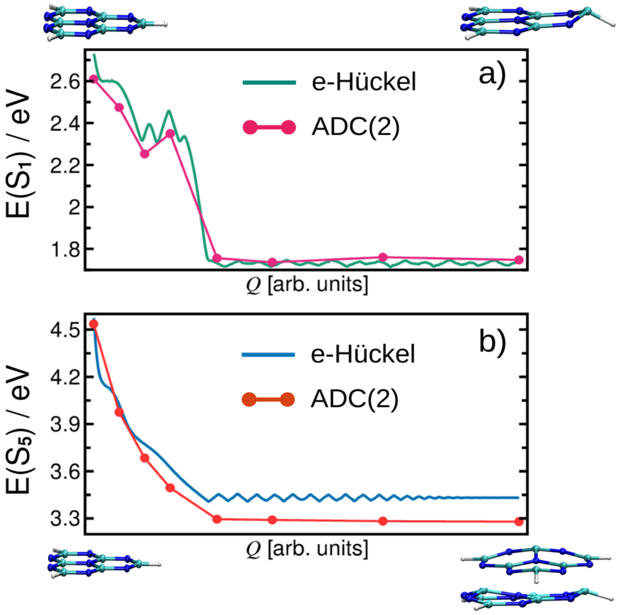

Figure S7: Excitation energies of the heptazine molecule calculated by the e-Hückel model and the ADC(2) method, for molecular geometries along the adiabatic relxation trajetories. The insets show the molecule's initial geometry (left) and its final geometry after stabilization in the excited-state (right).

The simulated annealing in the S_1 state was initiated from the ground-state geometry

and carried out with a phonon relaxation time of $\tau = 0.1$ fs, minimizing nuclear heating and reaching a final temperature of $T = 0.1$ K. In the S_5 state (Figure S7-b), the relaxation proceeds monotonically and involves an additional out-of-plane displacement of a bridging aromatic carbon atom. The agreement between the semiempirical excitation energies and the ADC(2) results is also satisfactory in this case.

As a final test, we compared the dynamical behavior of the excitation energies computed using the parametrized extended Hückel model with those obtained from the ADC(2) method. This comparison is based on molecular geometries sampled along excited-state molecular dynamics trajectories of the heptazine molecule, generated using the Ehrenfest-CSDM method. The results are presented in Figure S8.

In Figures S8-a to c, we present the molecular orbital energies as a function of time for an excited-state molecular dynamics simulation initiated by the photoexcitation of the heptazine molecule to the S_5 excited state (refer to the table in Figure S6-c). Figure S8-b shows snapshots of the heptazine molecule at $t = 0$, 0.04, and 0.08 ps along the nonadiabatic MD trajectory. For comparison with first-principles calculations, Figure S8-c presents Hartree–Fock single-point calculations for 11 molecular conformations equally spaced in time (dots), with the energies of the unoccupied molecular orbitals shifted downward to match the S_1 excitation energy of 2.7 eV at $t = 0$, as obtained from the ADC(2) method. The solid lines represent interpolation curves used for visualization purposes. The correspondence between Figures S8-a and S8-c demonstrates that the transitions between frontier orbitals in the heptazine molecule adequately reproduce the single-particle excitations calculated using ADC(2) throughout the nonadiabatic molecular dynamics, including at puckered geometries of the molecule. A similar comparison is shown in the right-hand panel (Figures S8-d to e) for an independent trajectory, also yielding satisfactory agreement.

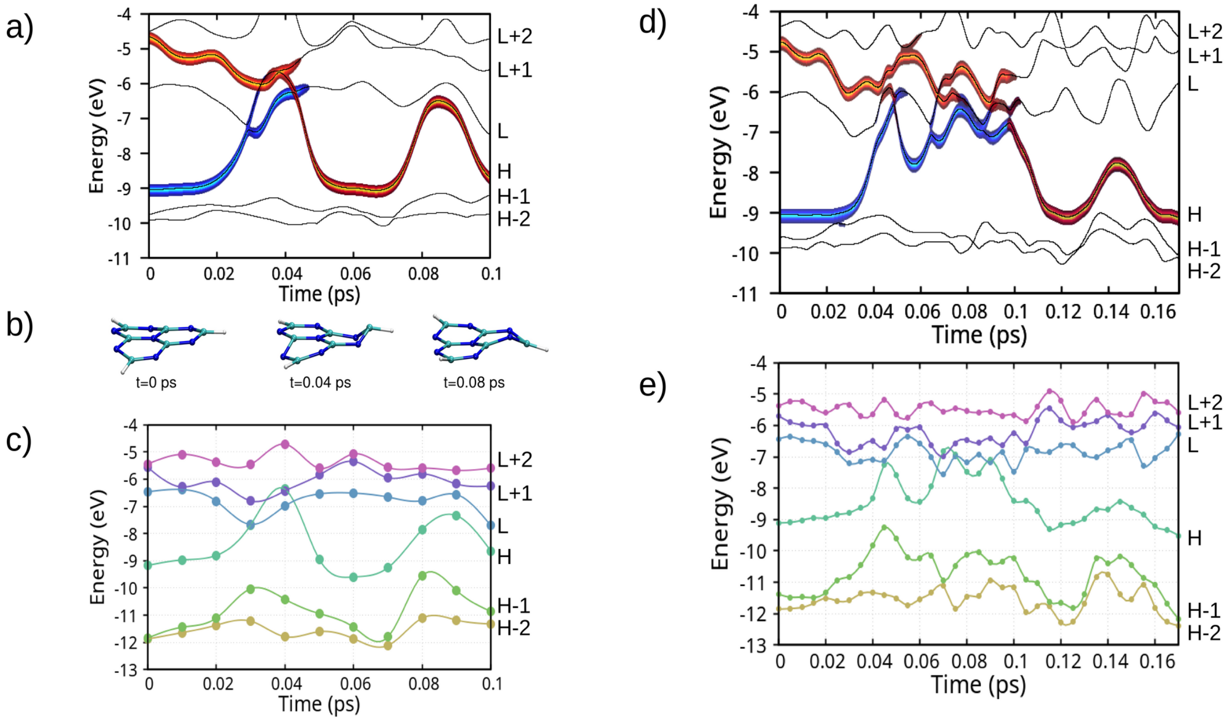

Figure S8: (a) Molecular orbital energies as a function of time for an excited-state molecular dynamics simulation of the heptazine (HTZ) molecule in the gas phase, performed using the Ehrenfest-CSDM method. The thick orange (blue) line indicates the occupation of the potential energy surfaces by the electron (hole) wavefunction. (b) Snapshots of the heptazine molecule at $t = 0$, 0.04, and 0.08 ps along the nonadiabatic trajectory. (c) Hartree–Fock single-point calculations for 11 molecular conformations equally spaced in time (dots), with the energies of the **unoccupied** molecular orbitals shifted downward to match the S_1 excitation energy of 2.7 eV at $t = 0$, as obtained by the ADC(2) method. The solid lines represent interpolation curves used for visualization purposes. (d)–(e) Same comparison shown for an independent trajectory, with similarly satisfactory agreement.

3.3 Water: Quantum Mechanical Model

The equilibrium structure of the water monomer in the S_0 state is characterized by $R_{O-H} = 0.97$ Å and $\angle HOH = 104.0^o$, which is reasonably well described by the TIP3P model used in this work.

On the other hand, the theoretical description of the thermodynamic and, notably, the electronic structure of water in the condensed phase remains an open challenge,[17–19] Hydrogen bonding plays a crucial role in shaping the electronic properties of liquid water, influencing both its structural organization and valence electronic states.[20–22] Unlike isolated water molecules, which can be accurately described using standard quantum chemistry approaches, the condensed phase introduces complex effects such as charge delocalization and polarization within the hydrogen-bonded network.[20,23] These strong intermolecular interactions lead to broadening and energy shifts in the electronic density of states, complicating the determination of a well-defined band gap. Standard electronic-structure methods, including hybrid functionals and non-self-consistent GW calculations, typically underestimate the band gap. More advanced self-consistent GW approaches provide a slightly improved picture but still exhibit significant uncertainties in the determination of ionization potential (IP) and electron affinity (EA), particularly due to the challenges in aligning these levels to the vacuum reference.[17–19]

A key difficulty in describing the electronic structure of liquid water arises from the inherent fluctuations of its hydrogen bond network and the dynamic polarization of surrounding solvent molecules. These fluctuations strongly influence redox properties and electronic excitations relevant to photochemical processes. Consequently, quantum mechanical/molecular mechanics (QM/MM) methods aiming to describe excited states in aqueous environments must carefully account for the number of explicitly treated water molecules.

Identifying these structures experimentally is challenging, and theoretical calculations face significant hurdles. One major difficulty is that commonly used density functional theory (DFT) methods suffer from self-interaction error,[24–26] making the total energy of the

species dependent on the exact exchange included in the functional.

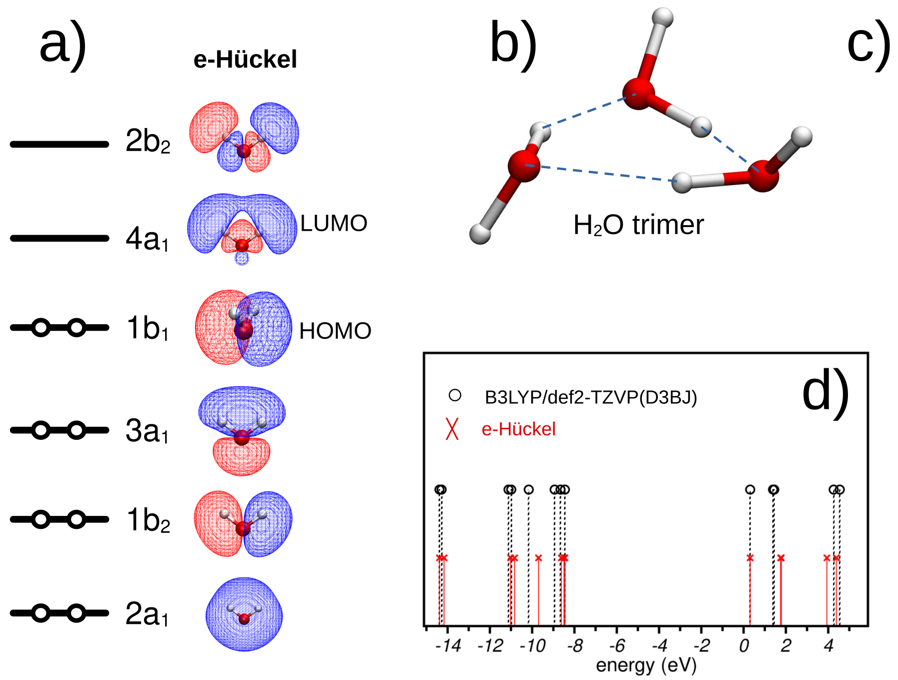

c)

	B3LYP/def2-TZVP(D3BJ)	e-Hückel
H-7	-14.3853	-14.33839
H-6	-14.2829	-14.1658
H-5	-11.1192	-11.0185
H-4	-10.9914	-10.8309
H-3	-10.1615	-9.6996
H-2	-8.9376	-8.6210
H-1	-8.6592	-8.4916
H	-8.4527	-8.4527
L	0.3125	0.3072
L+1	1.3898	1.7513
L+2	1.4504	1.7834
L+3	4.2587	3.9393
L+4	4.5483	4.3867

Figure S9: (a) Molecular orbitals of the H_2O monomer obtained using the parametrized extended Hückel model. (b) Water trimer employed in the parametrization process. (c) Molecular orbital energies (in eV) for the water trimer, computed using the DFT-B3LYP/def2-TZVP(D3BJ) method and the parametrized extended Hückel model. (d) Graphical representation of the MO energy data from (c).

To set the extended Hückel parameters for the water system in the present large-scale quantum mechanical dynamics calculations, we simultaneously considered the ground-state equilibrium structures of both the water monomer and a water trimer, as shown in Figure S9. The monomer was included to ensure that the molecular orbitals (MOs) retained the correct symmetry and that the dipole moment of the H_2O molecule in the gas phase was accurately reproduced. The trimer was incorporated to capture key hydrogen-bonding interactions and polarization effects. Reference energy levels for the H_2O trimer were computed using density functional theory (DFT). DFT calculations were carried out with the ORCA 5.0.4 package.[27] Structures were optimized with the wB97X-D3 functional[28] and the cc-pVTZ basis set. Auxiliary basis sets were set with the AutoAux tool[29] and dispersion corrections were used throughout all DFT calculations.[30] Time-dependent DFT (TD-DFT) calculations

were performed with the CAM-B3LYP functional[31] and the cc-pVDZ basis set.

The parametrization strategy simultaneously accounted for the monomer and trimer energies while preserving the dipole moment and orbital symmetries of gas-phase H_2O. Additionally, the electronic density of states was analyzed for 10 water droplets, each with a radius of $R = 10$ Å and containing approximately 125 water molecules on average. These droplets were extracted from larger thermalized water boxes at ambient conditions and were used to further refine the parameters by comparing the results with ab initio calculations[22,32] and experimental photoionization spectra.[20]

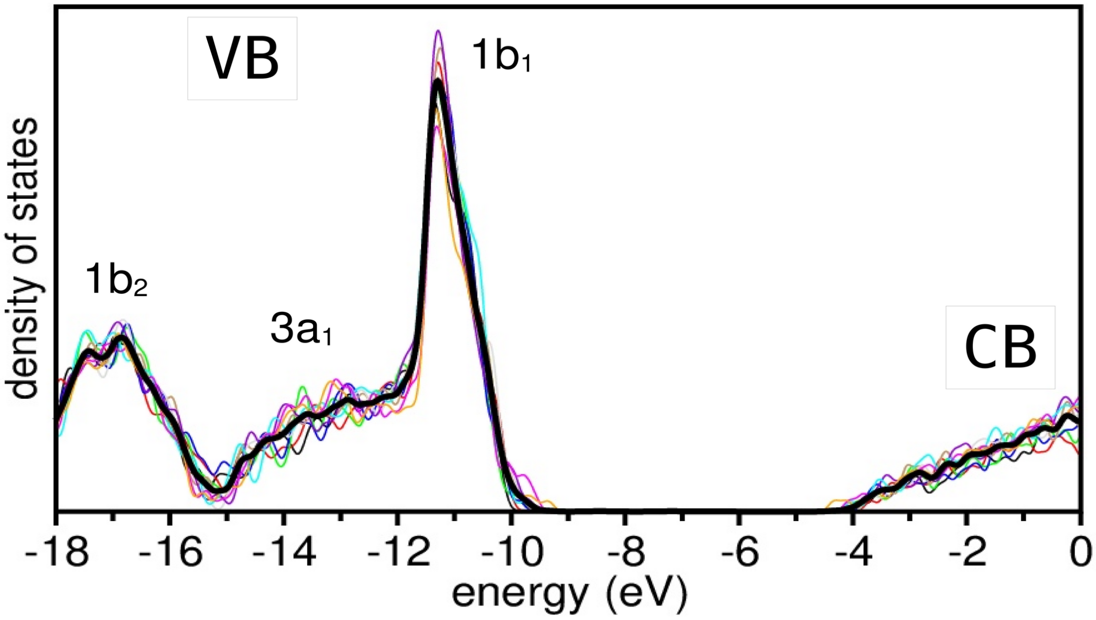

Figure S10: Electronic density of states (DOS) projected onto the water molecules of the HTZ-WAT system. The simulation box contains a total of 572 water molecules, with those within a droplet of radius $R = 10$ Å centered on the HTZ molecule treated quantum mechanically, while the remaining molecules form the MM region of the QM/MM system. The DOS is computed for 10 independent configurations of the HTZ-WAT system, each represented by a different color. The thick black line denotes the averaged DOS over all configurations. Labels indicate the conduction band (CB) and valence band (VB), with peaks corresponding to their respective molecular orbitals.

The optimized parameters for the water molecule are presented in Table S5. Notably, the additional p-orbital on the hydrogen (HW) atom is essential for achieving an accurate description of the water molecule. The parametrized extended Hückel model yields a gas-

phase dipole moment of 1.84 Debye for H_2O and an average dipole moment of 2.1 Debye for H_2O in the trimer structure.

Table S5: Optimized extended Hückel parameters for the water molecule.

Symbol	EHsymbol	residue	NoAt	Nvalen	n	spdf	V_i	zeta	K_i	V_{shift}
O	OW	WAT	8	6	2	s	-31.00012	2.099997	1.755990	3.35
O	OW	WAT	8	6	2	p	-14.66809	2.099997	3.627530	3.35
H	HW	WAT	1	1	1	s	-14.66685	0.667890	1.235250	3.35
H	HW	WAT	1	1	2	p	-0.52245	2.082434	0.010370	3.35

4 S1 Photorelaxation Dynamics Trajectories

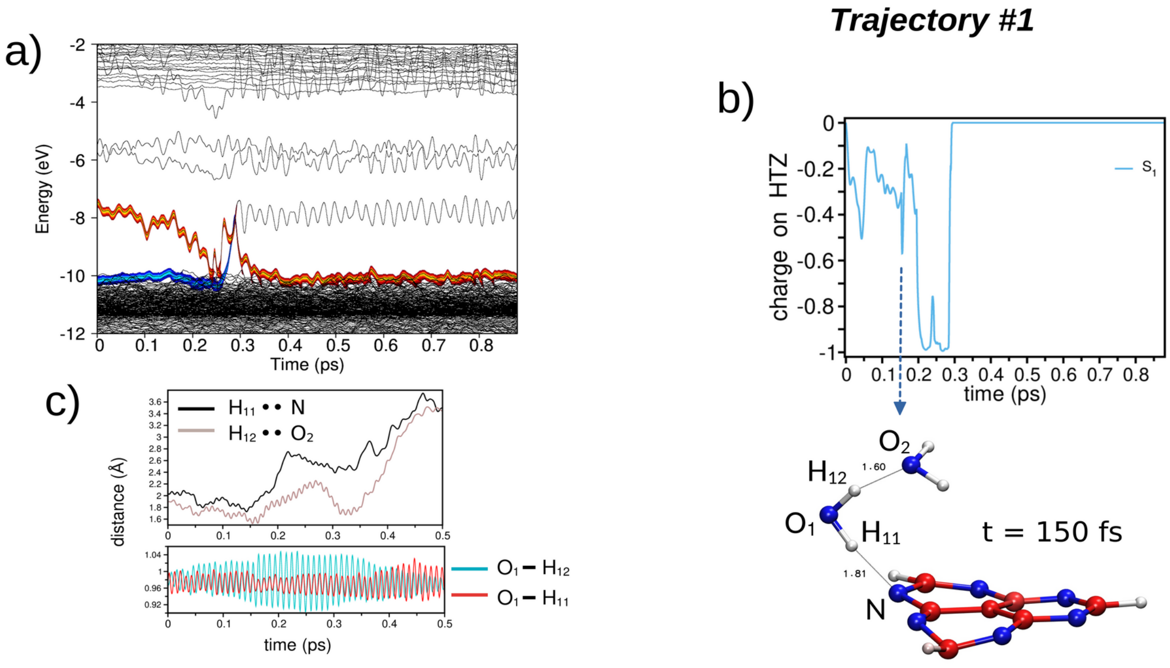

Figure S11: **Trajectory #1.**
(a) Photorelaxation dynamics of the electron–hole pair in solvated heptazine (HTZ) following excitation to the S_1 ($\pi \rightarrow \pi^*$) state.
(b) Net charge on HTZ as a function of time after S_1 excitation. Negative values indicate electron transfer from water to HTZ. The inset highlights a transient electron transfer event to HTZ at $t = 150$ fs, mediated by the $H_{11} \cdots N$ hydrogen bond.
(c) Time evolution of key interatomic distances relevant to the electron transfer process (see panel b).

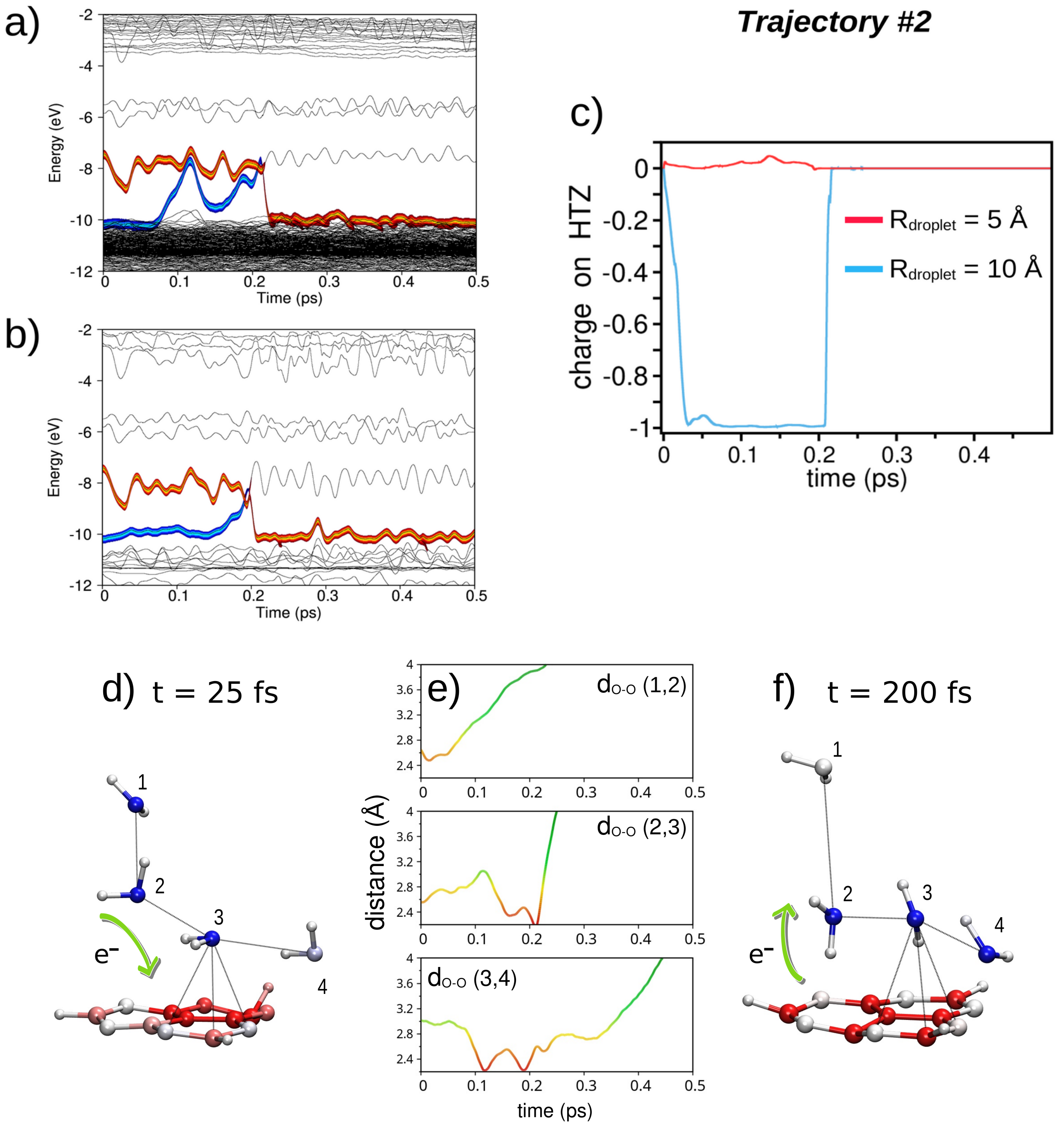

Figure S12: **Trajectory #2.**
(a) Photorelaxation dynamics of the electron–hole pair in fully solvated heptazine (HTZ) following excitation to the S_1 ($\pi \rightarrow \pi^*$) state, simulated with a quantum mechanical water droplet of radius $R_{\text{droplet}} = 10$ Å. (b) Same as (a), but for $R_{\text{droplet}} = 5$ Å.
(c) Net HTZ charge vs. time for $R_{\text{droplet}} = 5$ Å (red) and 10 Å (blue); negative values indicate electron transfer from water to HTZ, which is suppressed for smaller droplets. (d) Snapshot of water-to-HTZ electron transfer: red and blue regions show electron and hole densities; colorless atoms have no excess charge; water molecules are numbered 1–4. (e) O–O distances ($d_{\text{O-O}}$) with color scale: red = short, green = long. (f) Snapshot of electron back-transfer, reducing the cationic water cluster and driving recombination.

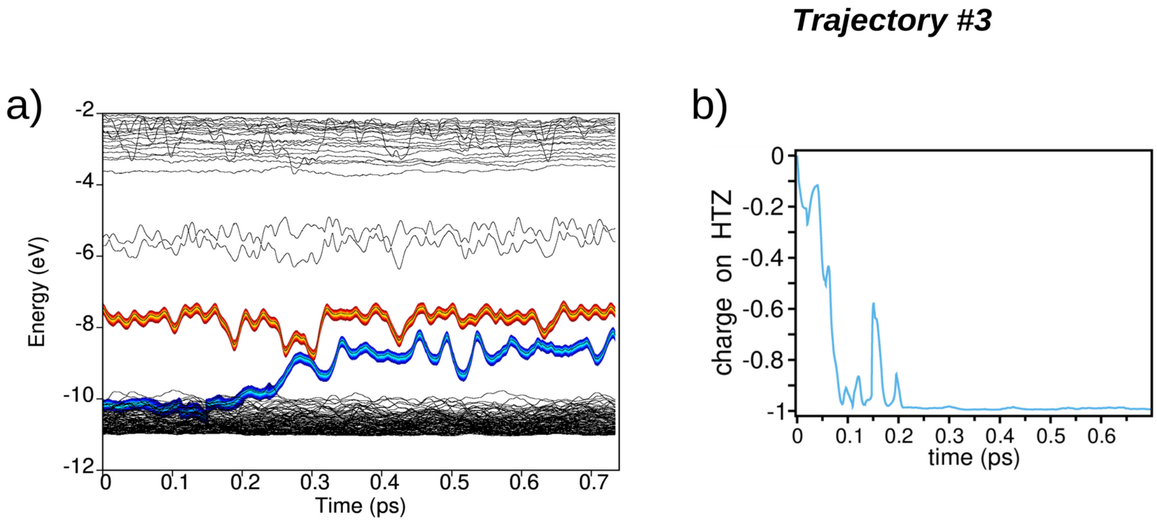

Figure S13: **Trajectory #3.**
(a) Photorelaxation dynamics of the electron–hole pair in solvated heptazine (HTZ) following excitation to the S_1 ($\pi \rightarrow \pi^*$) state. No electron–hole pair formation is observed within the simulation timescale.
(b) Net charge on HTZ as a function of time after S_1 excitation. Negative values indicate electron transfer from water to HTZ.

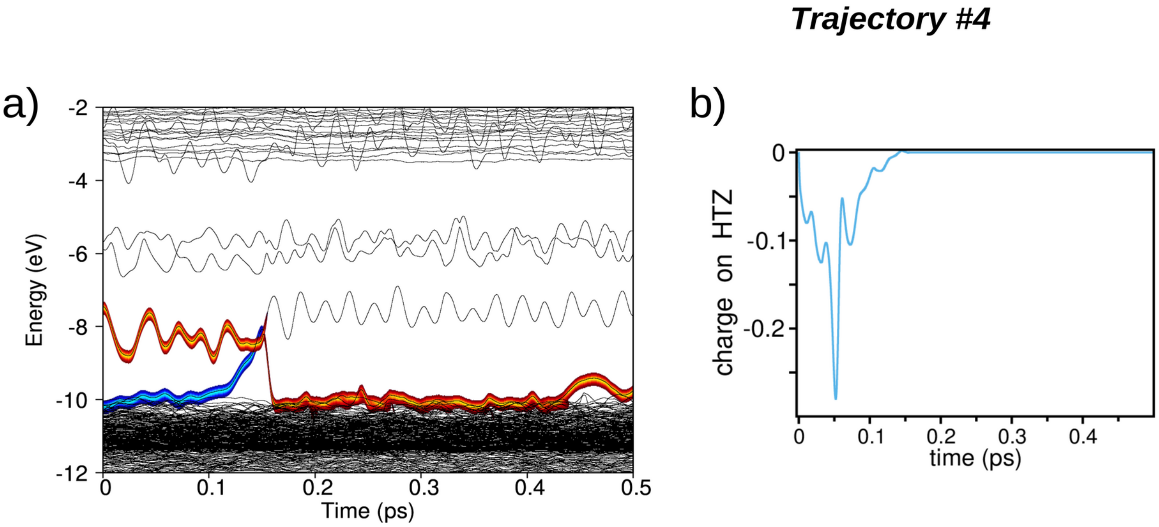

Figure S14: **Trajectory #4.**
(a) Photorelaxation dynamics of the electron–hole pair in solvated heptazine (HTZ) following excitation to the S_1 ($\pi \rightarrow \pi^*$) state.
(b) Net charge on HTZ as a function of time after S_1 excitation. Negative values indicate electron transfer from water to HTZ.

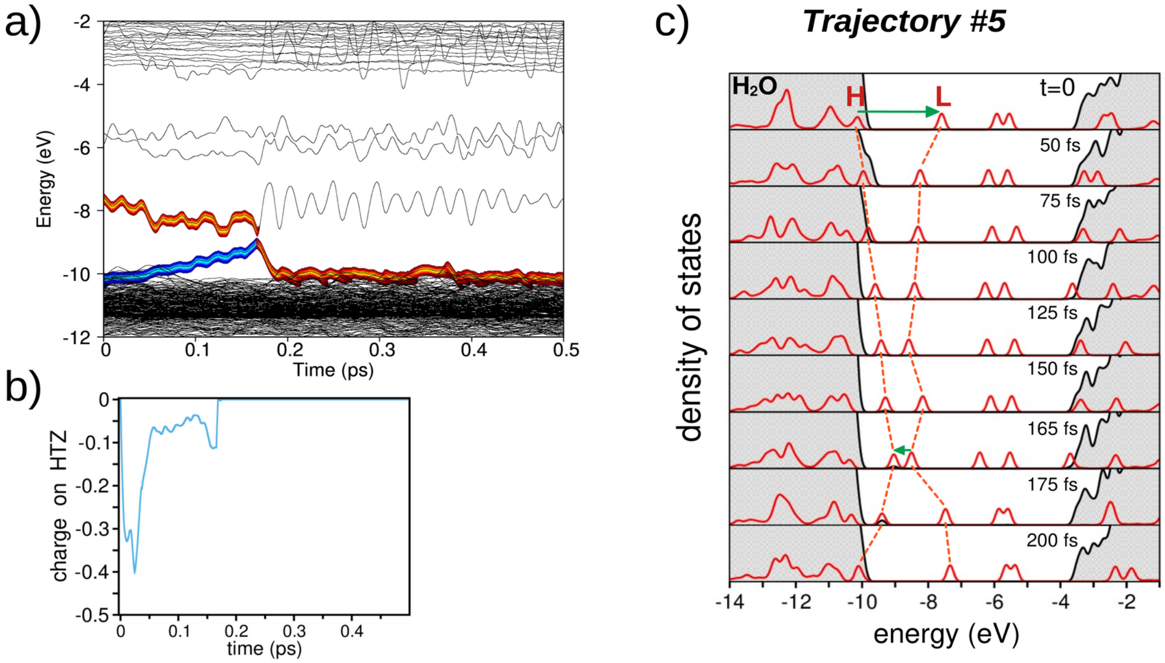

Figure S15: **Trajectory #5.**
(a) Photorelaxation dynamics of the electron–hole pair in solvated heptazine (HTZ) following excitation to the S_1 ($\pi \to \pi^*$) state.
(b) Net charge on HTZ as a function of time after S_1 excitation. Negative values indicate electron transfer from water to HTZ.
(c) Projected density of states (PDOS) for water (gray) and HTZ (red) over time, illustrating the internal conversion in the solvated HTZ. Energy levels are shown from $t = 0$ to 200 fs following photoexcitation (horizontal green arrow). The HOMO (H) and LUMO (L) of HTZ are labeled.

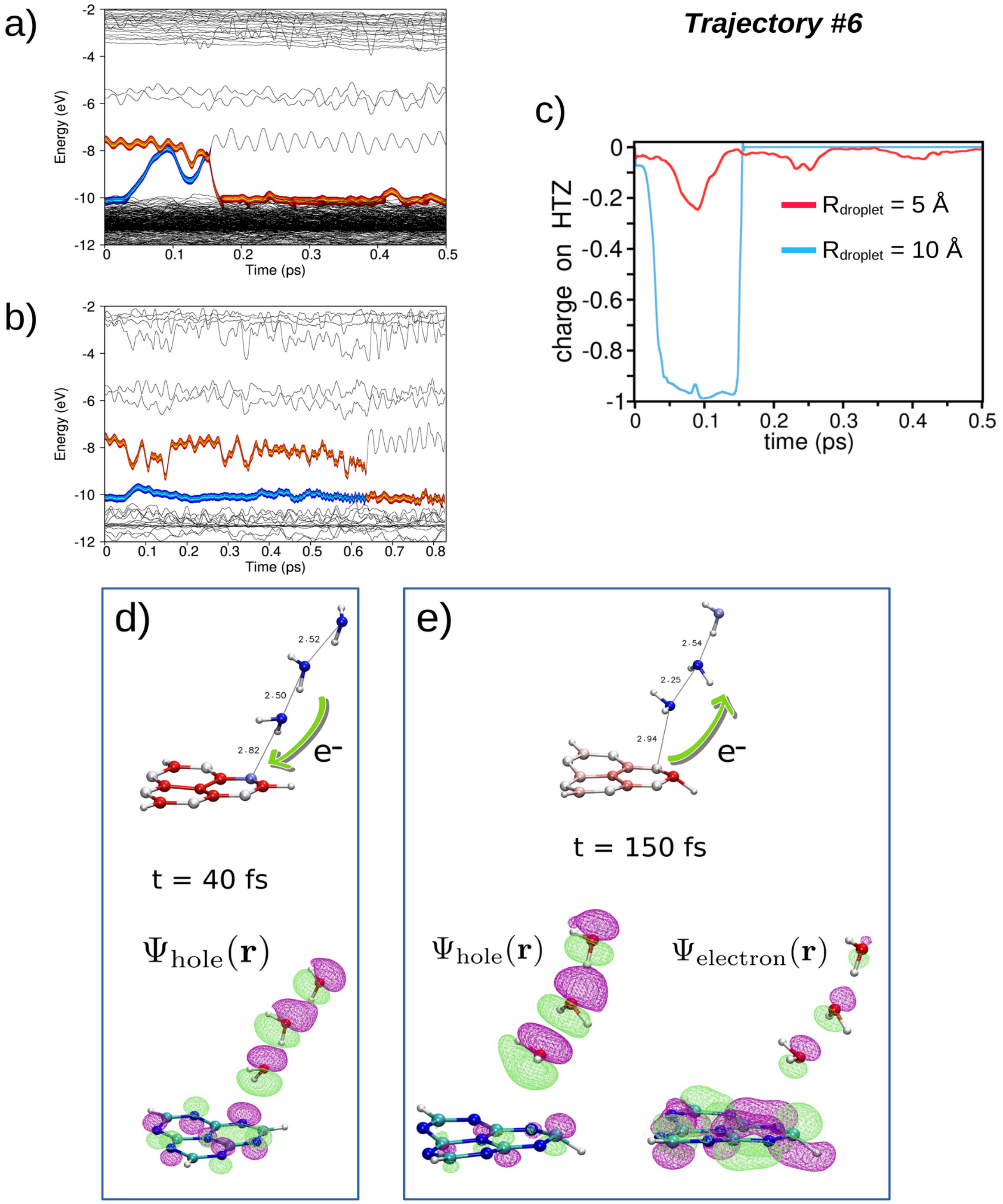

Figure S16: **Trajectory #6.**
(a) Photorelaxation dynamics of solvated heptazine (HTZ) following S_1 ($\pi \rightarrow \pi^*$) excitation with a quantum mechanical water droplet of radius $R_{\mathrm{droplet}} = 10$ Å. (b) Same as (a), but with $R_{\mathrm{droplet}} = 5$ Å. (c) Net charge on HTZ over time for $R_{\mathrm{droplet}} = 5$ Å (red) and 10 Å (blue). Negative values indicate electron transfer (ET) from water to HTZ; ET is suppressed in the smaller droplet. (d) Detail of ET event at $t = 40$ fs, showing the hole wavefunction. (e) Detail of back-ET at $t = 150$ fs, showing electron and hole wavefunctions. Panels (d) and (e) correspond to the $R_{\mathrm{droplet}} = 10$ Å system.

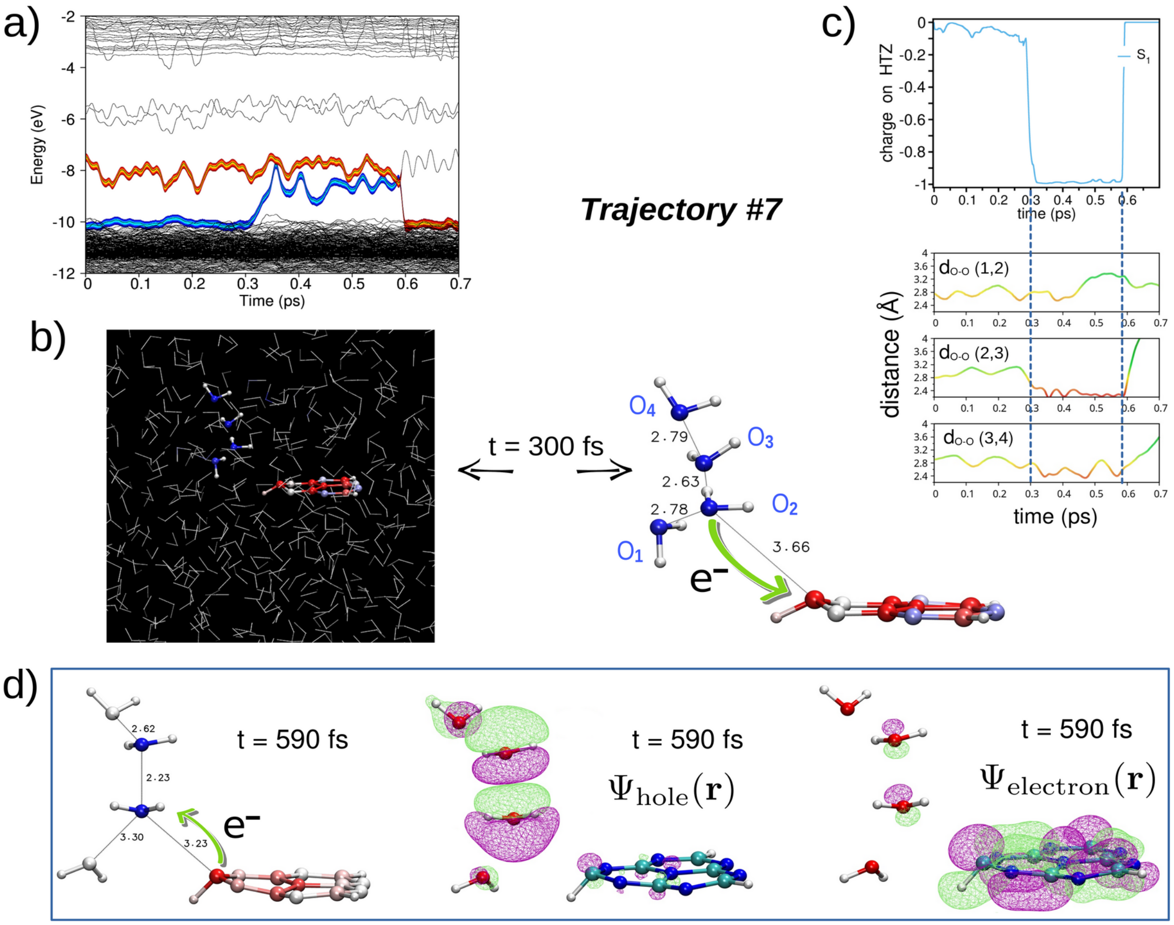

Figure S17: **Trajectory #7.**
(a) Photorelaxation dynamics of the electron–hole pair in solvated heptazine (HTZ) following excitation to the S_1 ($\pi \rightarrow \pi^*$) state.
(b) Picture of the electron transfer (ET) event from water to HTZ at t = 300 fs with a view of the water droplet.
(b) Net charge on HTZ as a function of time after S_1 excitation. Negative values indicate electron transfer from water to HTZ. Distance between oxygen atoms ($d_{\text{O-O}}$), with a color scale indicating short (red) and long (green) distances.
(d) Detail of the back-ET event from HTZ to water at $t = 590$ fs, showing hole and electron wavefunctions.

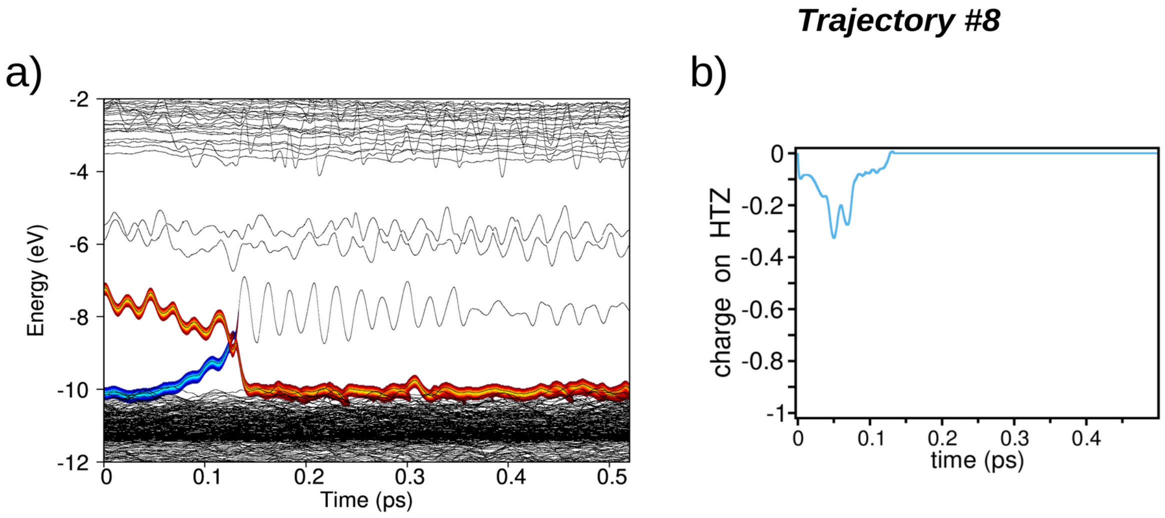

Figure S18: **Trajectory #8.**
(a) Photorelaxation dynamics of the electron–hole pair in solvated heptazine (HTZ) following excitation to the S_1 ($\pi \rightarrow \pi^*$) state.
(b) Net charge on HTZ as a function of time after S_1 excitation. Negative values indicate electron transfer from water to HTZ.

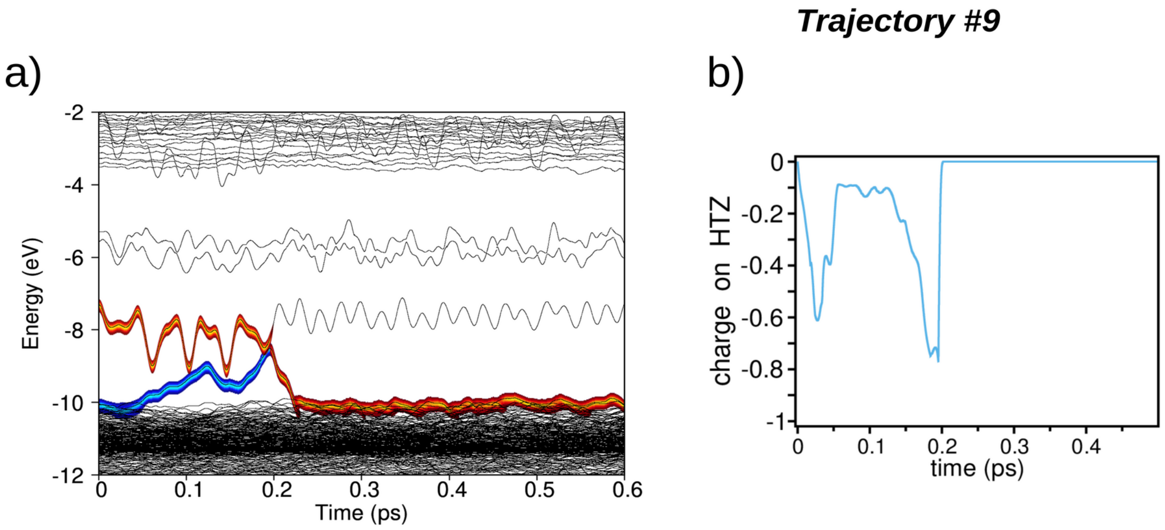

Figure S19: **Trajectory #9.**
(a) Photorelaxation dynamics of the electron–hole pair in solvated heptazine (HTZ) following excitation to the S_1 ($\pi \rightarrow \pi^*$) state.
(b) Net charge on HTZ as a function of time after S_1 excitation. Negative values indicate electron transfer from water to HTZ.

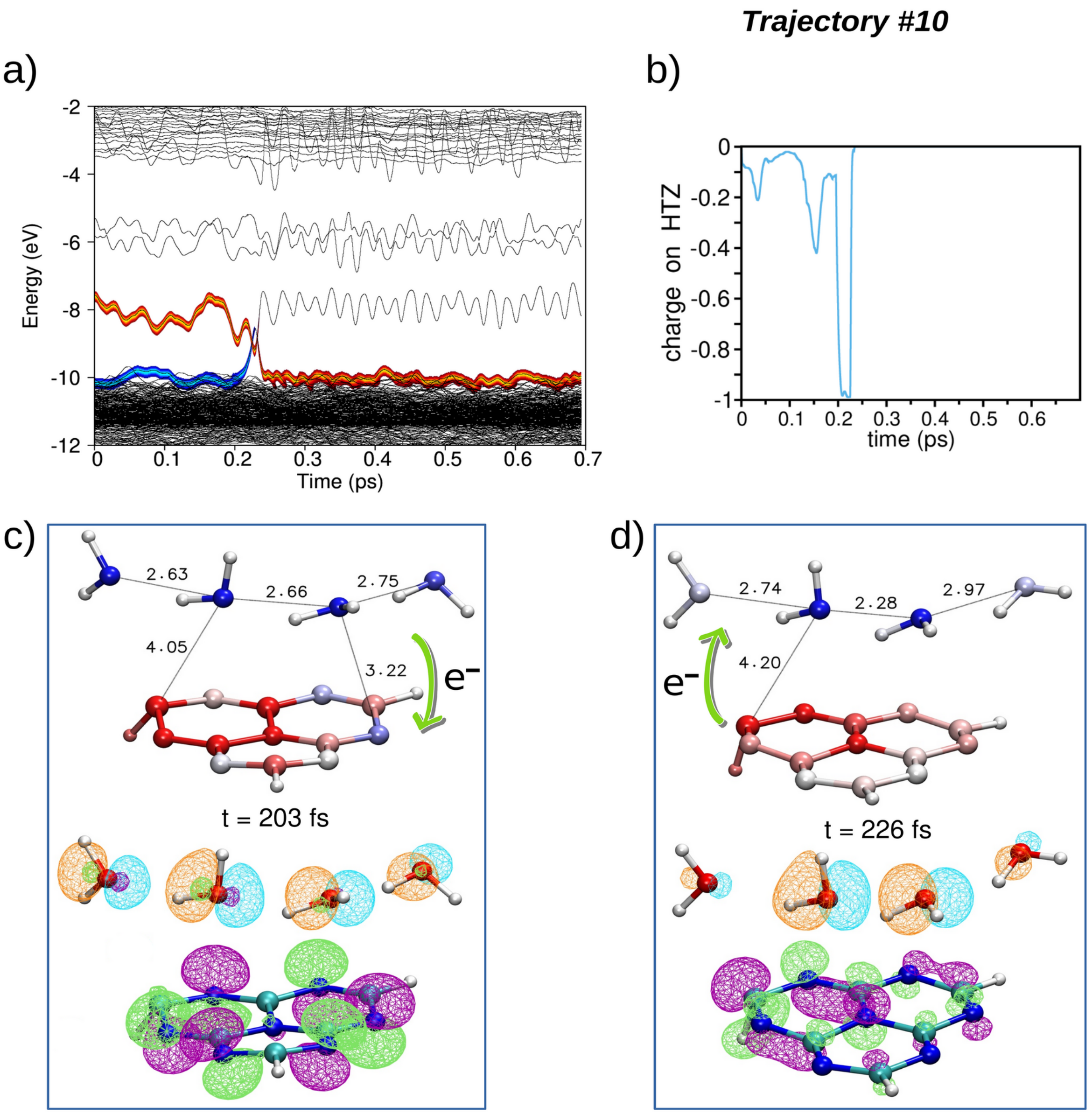

Figure S20: **Trajectory #10.**
(a) Photorelaxation dynamics of the electron–hole pair in solvated heptazine (HTZ) following excitation to the S_1 ($\pi \to \pi^*$) state.
(b) Net charge on HTZ as a function of time after S_1 excitation. Negative values indicate electron transfer from water to HTZ. (c) Detail of the electron transfer (ET) event at $t = 201$ fs, showing the hole wavefunction (orange/cyan) and electron wavefunction (green/purple).
(d) Same as in c) for the back-ET at $t = 226$ fs, showing electron and hole wavefunctions.

References

(1) Zhu, C.; Nangia, S.; Jasper, A. W.; Truhlar, D. G. Coherent switching with decay of mixing: An improved treatment of electronic coherence for non-Born–Oppenheimer trajectories. *J. Chem. Phys.* **2004**, *121*, 7658–7670.

(2) Zhu, C.; Jasper, A. W.; Truhlar, D. G. Non-Born-Oppenheimer Liouville-von Neumann Dynamics. Evolution of a Subsystem Controlled by Linear and Population-Driven Decay of Mixing with Decoherent and Coherent Switching. *J. Chem. Theory Comput.* **2005**, *1*, 527–540.

(3) Cheng, S. C.; Zhu, C.; Liang, K. K.; Lin, S. H.; Truhlar, D. G. Algorithmic decoherence time for decay-of-mixing non–Born–Oppenheimer dynamics. *J. Chem. Phys.* **2008**, *129*, 024112.

(4) Oliboni, R. S.; Bortolini, G.; Torres, A.; Rego, L. G. C. A nonadiabatic excited state molecular mechanics/extended Hückel Ehrenfest method. *J. Phys. Chem. C* **2016**, *120*, 27688–27698.

(5) Torres, A.; Prado, L. R.; Bortolini, G.; Rego, L. G. C. Charge Transfer Driven Structural Relaxation in a Push–Pull Azobenzene Dye–Semiconductor Complex. *J. Phys. Chem. Lett.* **2018**, *9*, 5926–5933.

(6) de Thieulloy, L.; Oliboni, R. S.; de Silva, P.; Rego, L. G. C. Aggregation Induced Effects on the Nonradiative Recombination Dynamics of Inverted Singlet–Triplet Heptazine-Based Materials. *J. Phys. Chem. A* **2025**, *129*, 5220–5233.

(7) Cornell, W. D.; Cieplak, P.; Bayly, C. I.; Gould, I. R.; Merz, K. M.; Ferguson, D. M.; Spellmeyer, D. C.; Fox, T.; Caldwell, J. W.; Kollman, P. A. A Second Generation Force Field for the Simulation of Proteins, Nucleic Acids, and Organic Molecules. *J. Am. Chem. Soc.* **1995**, *117*, 5179–5197.

(8) Rego, L. G. C.; Bortolini, G. Modulating the Photoisomerization Mechanism of Semiconductor-Bound Azobenzene-Functionalized Compounds. *J. Phys. Chem. C* **2019**, *123*, 5692–5698.

(9) Rego, L.G.C. DynEMol: tools for studying the Dynamics of Electrons in Molecules. https://github.com/lgcrego/Dynemol, 2018.

(10) Wang, J.; Wolf, R. M.; Caldwell, J. W.; Kollman, P. A.; Case, D. A. Development and testing of a general amber force field. *J. Comput. Chem.* **2004**, *25*, 1157–1174.

(11) Neria, E.; Fischer, S.; Karplus, M. Simulation of activation free energies in molecular systems. *J. Chem. Phys.* **1996**, *105*, 1902–1921.

(12) Mark, P.; Nilsson, L. Structure and Dynamics of the TIP3P, SPC, and SPC/E Water Models at 298 K. *J. Phys. Chem. A* **2001**, *105*, 9954–9960.

(13) Abraham, M. J.; Murtola, T.; Schulz, R.; Páll, S.; Smith, J. C.; Hess, B.; Lindahl, E. GROMACS: High performance molecular simulations through multi-level parallelism from laptops to supercomputers. *SoftwareX* **2015**, *1-2*, 19–25.

(14) Ammeter, J. H.; Buergi, H. B.; Thibeault, J. C.; Hoffmann, R. Counterintuitive orbital mixing in semiempirical and ab initio molecular orbital calculations. *J. Am. Chem. Soc.* **1978**, *100*, 3686–3692.

(15) Balasubramani, S. G.; Chen, G. P.; Coriani, S.; Diedenhofen, M.; Frank, M. S.; Franzke, Y. J.; Furche, F.; Grotjahn, R.; Harding, M. E.; Hättig, C. et al. TURBOMOLE: Modular program suite for ab initio quantum-chemical and condensed-matter simulations. *J. Chem. Phys.* **2020**, *152*, 184107.

(16) Plasser, F.; Crespo-Otero, R.; Pederzoli, M.; Pittner, J.; Lischka, H.; Barbatti, M. Surface Hopping Dynamics with Correlated Single-Reference Methods: 9H-Adenine as a Case Study. *J. Chem. Theory Comput.* **2014**, *10*, 1395–1405.

(17) Cabral do Couto, P.; Chipman, D. M. Insights into the ultraviolet spectrum of liquid water from model calculations. *J. Chem. Phys.* **2010**, *132*, 244307.

(18) Gaiduk, A. P.; Pham, T. A.; Govoni, M.; Paesani, F.; Galli, G. Electron affinity of liquid water. *Nat. Commun.* **2018**, *9*, 247.

(19) Ambrosio, F.; Guo, Z.; Pasquarello, A. Absolute Energy Levels of Liquid Water. *J. Phys. Chem. Lett.* **2018**, *9*, 3212–3216.

(20) Winter, B.; Weber, R.; Widdra, W.; Dittmar, M.; Faubel, M.; Hertel, I. V. Full Valence Band Photoemission from Liquid Water Using EUV Synchrotron Radiation. *J. Phys. Chem. A* **2004**, *108*, 2625–2632.

(21) Cruzeiro, V. W. D.; Wildman, A.; Li, X.; Paesani, F. Relationship between Hydrogen-Bonding Motifs and the 1b1 Splitting in the X-ray Emission Spectrum of Liquid Water. *J. Phys. Chem. Lett.* **2021**, *12*, 3996–4002.

(22) Chen, W.; Ambrosio, F.; Miceli, G.; Pasquarello, A. Ab initio Electronic Structure of Liquid Water. *Phys. Rev. Lett.* **2016**, *117*, 186401.

(23) Elles, C. G.; Rivera, C. A.; Zhang, Y.; Pieniazek, P. A.; Bradforth, S. E. Electronic structure of liquid water from polarization-dependent two-photon absorption spectroscopy. *J. Chem. Phys.* **2009**, *130*, 084501.

(24) Marsalek, O.; Elles, C. G.; Pieniazek, P. A.; Pluhařová, E.; VandeVondele, J.; Bradforth, S. E.; Jungwirth, P. Chasing charge localization and chemical reactivity following photoionization in liquid water. *J. Chem. Phys.* **2011**, *135*, 224510.

(25) Chipman, D. M. Hemibonding between Water Cation and Water. *J. Phys. Chem. A* **2016**, *120*, 9618–9624.

(26) Rana, B.; Herbert, J. M. Hidden Hemibonding in the Aqueous Hydroxyl Radical. *J. Phys. Chem. Letters* **2021**, *12*, 8053–8060.

(27) Neese, F. Software update: The ORCA program system—Version 5.0. *WIREs Comput. Mol. Sci.* **2022**, *12*, e1606.

(28) Lin, Y.-S.; Li, G.-D.; Mao, S.-P.; Chai, J.-D. Long-Range Corrected Hybrid Density Functionals with Improved Dispersion Corrections. *J. Chem. Theory Comput.* **2013**, *9*, 263–272.

(29) Stoychev, G. L.; Auer, A. A.; Neese, F. Automatic Generation of Auxiliary Basis Sets. *J. Chem. Theory Comput.* **2017**, *13*, 554–562.

(30) Grimme, S.; Antony, J.; Ehrlich, S.; Krieg, H. A consistent and accurate ab initio parametrization of density functional dispersion correction (DFT-D) for the 94 elements H-Pu. *J. Chem. Phys.* **2010**, *132*, 154104.

(31) Yanai, T.; Tew, D. P.; Handy, N. C. A new hybrid exchange–correlation functional using the Coulomb-attenuating method (CAM-B3LYP). *Chem. Phys. Lett.* **2004**, *393*, 51–57.

(32) Ehrmaier, J.; Domcke, W.; Opalka, D. Mechanism of Photocatalytic Water Oxidation by Graphitic Carbon Nitride. *J. Phys. Chem. Lett.* **2018**, *9*, 4695–4699.
